# The evolution of plant phenomics: global insights, trends, and collaborations (2000-2021)

**DOI:** 10.3389/fpls.2024.1410738

**Published:** 2024-07-22

**Authors:** Lana Awada, Peter W. B. Phillips, Ana Maria Bodan

**Affiliations:** ^1^ Centre for the Study of Science and Innovation Policy, Johnson Shoyama Graduate School of Public Policy, University of Saskatchewan, Saskatoon, SK, Canada; ^2^ Canadian Hub for Applied and Social Research, University of Saskatchewan, Saskatoon, SK, Canada

**Keywords:** plant phenomics, agricultural innovation, geographic dynamics, publications, patents, networks, collaborations, social network analysis

## Abstract

**Introduction:**

Phenomics, an interdisciplinary field that investigates the relationships between genomics and environmental factors, has significantly advanced plant breeding by offering comprehensive insights into plant traits from molecular to physiological levels. This study examines the global evolution, geographic distribution, collaborative efforts, and primary research hubs in plant phenomics from 2000 to 2021, using data derived from patents and scientific publications.

**Methods:**

The study utilized data from the EspaceNet and Lens databases for patents, and Web of Science (WoS) and Scopus for scientific publications. The final datasets included 651 relevant patents and 7173 peer-reviewed articles. Data were geocoded to assign country-level geographical coordinates and underwent multiple processing and cleaning steps using Python, Excel, R, and ArcGIS. Social network analysis (SNA) was conducted to assess collaboration patterns using Pajek and UCINET.

**Results:**

Research activities in plant phenomics have increased significantly, with China emerging as a major player, filing nearly 70% of patents from 2010 to 2021. The U.S. and EU remain significant contributors, accounting for over half of the research output. The study identified around 50 global research hubs, mainly in the U.S. (36%), Western Europe (34%), and China (16%). Collaboration networks have become more complex and interdisciplinary, reflecting a strategic approach to solving research challenges.

**Discussion:**

The findings underscore the importance of global collaboration and technological advancement in plant phenomics. China's rise in patent filings highlights its growing influence, while the ongoing contributions from the U.S. and EU demonstrate their continued leadership. The development of complex collaborative networks emphasizes the scientific community's adaptive strategies to address multifaceted research issues. These insights are crucial for researchers, policymakers, and industry stakeholders aiming to innovate in agricultural practices and improve crop varieties.

## Introduction

Plant breeding is currently being transformed by advancements in understanding the plant’s phenotype, the complex interplay between plant genetic makeup and the physical environment, encompassing both above- and below-ground factors, throughout its growth and development. This interaction affects not only the growth and development process of plants, which can be measured by the structural traits at the cellular, tissue, organ, and plant levels, but also the plant’s functioning, which can be measured by the physiological traits. These traits have an impact on the plant’s productivity and performance, including their morphology, accumulated biomass, commercial yield, and resource utilization efficiency (Zhao et al., 2019). Given the challenges posed by population growth, climate change, and resource shortages in ensuring food, feed, and fiber supply, improving the understanding and performance of structural and physiological traits has become of paramount importance.

The rapid progress in genetic analysis techniques and the expanding size of plant populations have made phenotyping a bottleneck in understanding the genetic basis of complex traits (Song et al., 2021). The development of molecular technologies, such as high-throughput DNA marker genotyping and next-generation sequencing, has enabled plant genotype analysis at a reduced cost, leading to improved understanding of the genetic architecture of traits. However, identifying the molecular basis of complex quantitative traits (QTs) – such as yield, stress tolerance, and resource and input utilization efficiency – remains elusive.^1^ Quantitative traits are governed by many genes, known as polygenes, resulting in continuous phenotypic variation due to their low inheritance and the influence of environmental factors (Awada et al., 2018).^2^ Therefore, to fully comprehend the genetic basis of QTs, it is essential to couple and integrate genomic data with reliable, precise, and high-throughput phenotyping and environmental information (Thomas, 2010; Nakaya and Isobe, 2012; Singh and Singh, 2015). This integration enables the identification of molecular markers associated with traits and facilitates quantitative trait locus (QTL) mapping and genome-wide association studies (GWAS) (Yang et al., 2020). These tools can then be utilized to enhance the efficiency of new breeding techniques like genomic selection and genome editing, leading to a shorter breeding cycle, increased genetic gain, and the development of improved crop varieties (Thomson et al., 2022).^3^

Recent advances in high-throughput phenotyping techniques, driven by interdisciplinary research collaborations, have led to the emergence of a new research paradigm called “phenomics”. This field integrates biology, bioinformatics, computer science, engineering, and statistics to achieve a comprehensive understanding of complex plant traits at the molecular and physiological levels (Zhao et al., 2019). This state-of-the-art approach facilitates the acquisition and analysis of high-throughput and multi-dimensional phenotypic data during plant growth in complex environments, at a genome-wide scale. It aims to provide precise, reliable, and extensive data on plant architecture, composition, and growth at different scales. Phenotypic data are mainly collected using non-invasive and non-destructive imaging (i.e., RGB, chlorophyll fluorescence, hyperspectral, thermal, and lidar imaging), spectroscopy, remote sensing, robotics, and automation technologies (Yang et al., 2020)^4^. Phenomics combines high-performance computing and artificial intelligence technologies to effectively analyze diverse phenotyping information and physical and biochemical traits of plants, and to develop comprehensive tools to integrate with other omics data (i.e., genomics, transcriptomics, proteomics, metabolomics) (Zhao et al., 2019).

The objective of the paper is to provide a comprehensive analysis of the evolution, distribution, and impact of global plant phenomics research from 2000 to 2021. By examining patent and scientific publication data, this study aims to uncover the geographic and temporal patterns of innovation within the field. We seek to identify the key contributors, collaborative networks, and innovation hotspots that have shaped the landscape of plant phenomics. By offering insights into the current landscape and potential future directions of this research area, the study underscores the importance of interdisciplinary approaches, technological advancements, and global collaboration in facing challenges related to agriculture and food security. The findings are expected to serve as a valuable resource for researchers, policymakers, and industry stakeholders who are interested in the development and future prospects of plant phenomics, in particular, and agricultural innovation, in general.

## Advancements in plant phenomics: a review

Different phenotyping approaches capture different levels of detail and complexity of plant traits. For instance, automated phenotyping platforms in controlled environments and high-throughput methodologies in the field are designed to collect a large amount of data quickly, prioritizing high-throughput capture and assessment. On the other hand, phenotyping at the organ, tissue, and cellular level aims to obtain detailed information about individual plant components, such as leaves or roots, emphasizing in-depth phenotyping with higher resolution (Dhondt et al., 2013). The choice of phenotyping approach depends on the research question and the required level of detail. The integration of these technologies with automatic control technology, computers, robotics, and aeronautics has led to the development of an increasing number of high-throughput phenotyping platforms for investigating crop phenotypic traits (Zhao et al., 2019; Song et al., 2021).

These platforms are divided into three types based on the imaging level: microscopic, ground-based, and aerial phenotyping platforms, which allow the characterization of phenotypic traits at the tissue, organ, individual plant, plot, and field levels. Microscopic phenotyping platforms capture detailed information about individual plant components, such as leaves or roots, at a high resolution, while ground-based phenotyping platforms provide detailed information about individual plants, such as plant height, biomass, and leaf area index. Aerial phenotyping platforms, such as drones or satellites, provide information on large-scale plant phenotypes, such as crop yield and stress responses over large areas (Song et al., 2021).

In recent years, there has been a significant effort to develop high-throughput phenotyping techniques for various targets, ranging from cells to canopy. For cells and tissues, high-resolution imaging techniques like micro-computed tomography and microscopic imaging have been used to determine cellular properties such as cell structure, growth rate, and tissue morphology (Du et al., 2016). Visible light imaging has been used to assess morphological traits like color, length of coleoptile, and germination rate of seeds, while X-ray imaging has been used to evaluate seed morphometric features and tissue integrity (Ma et al., 2016; Zhang and Zhang, 2018; Zhang et al., 2018). Near-infrared spectroscopy and time-domain pulsed nuclear magnetic resonance have demonstrated advantages in determining the content of protein, oil, and fatty acids in seeds (Anderson et al., 2019). High-throughput phenotyping techniques have also been documented for individual plants and canopy, with various sensors and platforms deployed to obtain phenotypes at these scales. These techniques have been used to assess plant morphological, physiological, and pathological traits, particularly in the evaluation of abiotic stress, pest stress, and yield quality in crops (Zhang et al., 2018). Furthermore, the potential applications of high-throughput phenotyping techniques in disease assessment have been detailed, along with hyperspectral imaging and three-dimensional sensing for plant phenotyping (Liu et al., 2020). The use of unmanned aerial vehicles for field crop phenotyping has also been reviewed, with a focus on deployed sensors and their characteristics (Zhao et al., 2019; Xiao et al., 2022).

The utilization of robust high-throughput phenotyping techniques permits the continual imaging of plants at brief intervals, which in turn facilitates efficient analysis of all activities involved in plant growth. These techniques entail the utilization of image processing algorithms to extract traits from high-resolution images of plant samples, which are then employed to calculate derived parameters such as the height/width ratio (Xiao et al., 2022). The application of machine learning algorithms, specifically deep learning, has exhibited significant promise in plant phenotyping research (Mochida et al., 2019). Studies have demonstrated success in a wide range of plant phenotyping tasks using deep convolutional neural networks (CNNs) in various vision-based computer problems, including detecting, diagnosing and classifying fruits and flowers, and counting leaf numbers (LeCun et al., 2015). The achievement of these tasks was made possible by the massive amounts of captured and annotated plant images. From a machine vision perspective, deep learning has become an essential framework technique in image-based plant phenotyping. In the broad category of machine learning techniques, deep learning demonstrates advantages in many image-based tasks, such as object detection and localization, semantic segmentation, and image classification, without requiring feature description and extraction procedures (LeCun et al., 2015; Mohanty et al., 2016). With the promising results achieved thus far, it is expected that deep learning will continue to play a prominent role in breaking through the bottlenecks of plant phenotyping (Zhao et al., 2019; Xiao et al., 2022).

Despite the significant progress made in high-throughput plant phenotyping, several challenges remain to be addressed to fully exploit the potential of phenomics for crop improvement and breeding. One of the most significant challenges is feature extraction, which involves the identification of relevant phenotypic traits from the large and complex datasets generated by high-throughput phenotyping. Developing effective methods to extract features is crucial for advancing image-based phenotyping and facilitating association analysis in the era of omics (Yang et al., 2020). Another critical challenge is data management, which includes data storage, sharing, and analysis. As the amount of phenotypic data generated by high-throughput phenotyping continues to increase, advanced data management tools are necessary to support data integration, interoperability, ontologies, shareability, and globality.

Awada et al. (2021) presented a comprehensive framework for phenotypic data governance and stewardship, emphasizing the use of FAIR principles, MIAPPE standards, and crop ontology as guidelines for managing phenotypic data. However, the development of big-data management tools capable of handling the complexity and diversity of phenotypic data remains necessary. Data mining is also a significant challenge in plant phenomics. The large amount of phenotypic data generated by high-throughput phenotyping requires powerful data mining techniques such as dynamic growth models, support vector machines, and deep neural networks to extract meaningful information. These techniques can detect complex patterns and relationships in the data, allowing researchers to identify key traits of interest for target environments and streamline the process of genotype-to-phenotype integration. However, there are scientific and technical challenges that need to be addressed, such as the validity and practicality of the models and their interactions with the complex G × E × M interactions in plant phenotyping. Finally, feature preprocessing is a crucial step in data analysis that involves outlier detection, normalization, correlation analysis, and heritability analysis. Developing effective preprocessing methods is essential to ensure the accuracy and reliability of the phenotypic data used in subsequent analyses. For example, outlier detection is necessary to identify and remove anomalies from the data, while normalization is crucial to transform data into a standard scale to facilitate comparison (Yang et al., 2020).

All these evolving and emerging techniques involve interdisciplinary investigations in teams, both within and between countries. The rest of this paper investigates the evolving landscape of global innovation in digital plant phenomics.

## Research method

This investigation focuses on 21-year period, divided into two distinct phases – 2000-2010 and 2011-2021 – using a systematic approach to collect and analyze a combination of peer-reviewed publications and patent data.

For patent data, the EspaceNet database was our primary source, owing to its comprehensive coverage, ability to compile patent families, and advanced search functionalities. In the second stage of patent data collection, we utilized the Lens platform to access additional metadata (i.e. citation data). We initially retrieved 688 patents. However, after several rounds of screening for relevancy, we excluded 37 patents that did not align with our research criteria, resulting in a final dataset of 651 records (refer to Appendix B for query syntax details). For international-scale analysis, we used geocoding to assign country-level geographical coordinates to patents. This country-level approach was adopted due to the inconsistent availability of detailed geographical information, like inventors’ addresses, in certain national databases.

For scientific publication data, we used two academic databases – Web of Science (WoS) and Scopus. This choice was driven by their extensive coverage. We utilized the Bibliometrix R Package’s mergeBdSource function to merge the data from these databases and remove any duplicates. Furthermore, we conducted trial searches and compared the outcomes, in addition to analyzing the metadata, to ensure our ability to extract relevant data and fields that could be integrated across both databases. We performed queries on each database using the search terms and criteria detailed in Appendix B. This process yielded a final dataset of 7,173 peer-reviewed articles from the years 2000 to 2021. Additionally, for analyses on an international scale, we determined research locations based on the authors’ affiliations, applying geocoding at the levels of postal code, sub-city, and city.

The datasets underwent multiple processing and cleaning procedures using Python, Excel, R, and ArcGIS. Initially, Python was employed to parse and clean metadata fields. Subsequently, in Excel, we retrieved missing information and manually entered it, standardizing the names of authors, applicants, organizations, and the names of countries and cities. This stage also included the creation of additional variables, such as organization type, and further data cleaning, validation, and verification to ensure data accuracy and reliability.

For social network analysis, R was utilized to restructure the datasets. Pajek and UCINET were used for data processing and visualization. In the context of patent data, networks like co-applicant, co-inventor, and co-location were generated to identify collaboration patterns over time among various organizations and countries. Likewise, for the scientific publication dataset, networks such as co-authorship, co-affiliation, and co-location were created and analyzed at both city and country levels.

The preparation of thematic maps at the city and country levels involved several steps, including verifying that the dataset contained all necessary information for geocoding. Data cleaning was performed to correct inconsistent city and county names using geocoding reference data. ArcGIS Online was utilized for city-level mapping to calculate geographical coordinates, while country-level data was integrated with a spatial layer from Esri Maps (2023) to depict collaboration networks. Collaboration networks between countries were illustrated using the location of each country’s capital city. The map layout was designed in ArcMap 10.8.1, where publication counts, national collaborations, and international collaborations were displayed using a manual classification scheme.

To examine the changes over two decades, we employed a range of statistical methods, including T-tests, the Chi-Square Test of Homogeneity, and the Wilcoxon-Mann-Whitney Test, using SPSS 28. Furthermore, R, UCINET and Pajek were used for visualizing collaboration networks between countries and conducting network analyses. Specifically, we calculated centrality metrics across various networks to assess the centrality of inventors and applicants in patent networks, as well as authors and their affiliated organizations in publication networks. We also measured network cohesion to explore shifts in collaboration patterns over time and space. We identified key hotspots by considering several factors, such as the number of collaborations, the volume of publications, and the presence of active researchers and institutions, whether at the city or country level.

## The global landscape of phenomics innovation: a two-decade review of patents and scientific papers

During the 2000 and 2021 period a significant increase in phenomics-related patents and publications can be noted, with a shift in innovation leadership from the US and Europe to China, marked by China’s dominance in patent filings despite ongoing concerns about patent quality. Between 2000 and 2021, 7,173 scientific papers were published in the area of digital agricultural phenotyping, 20% between 2000 and 2010 and 80% in the following decade. Over the same period, 651 patents were retrieved, with 16% filed between 2000 and 2010, and 84% between 2011 and 2021.

**Figure 1:**
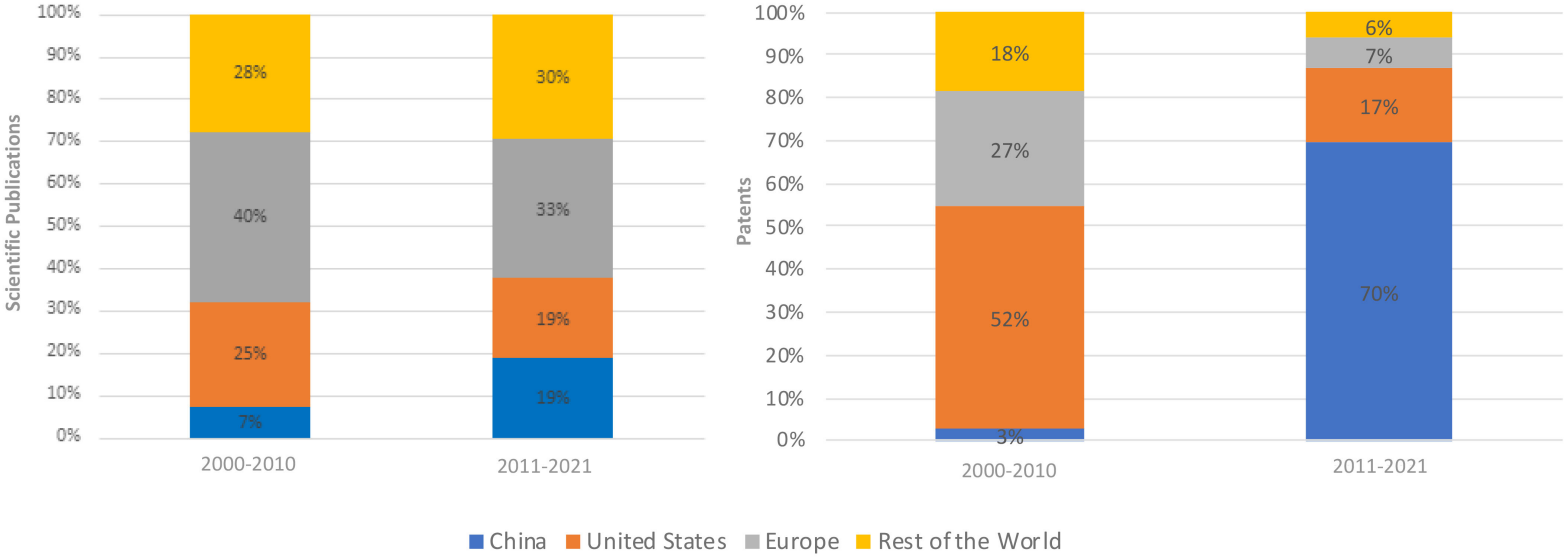
Global evolution of scientific publications (left) and patents (right).

From 2000 to 2010, the U.S. and Europe – notably Germany, France, and the UK – played a leading role, contributing to 65% of global scientific publications and accounting for 79% of all patents (**Figure 1**). The rest of the world, particularly Australia, Canada, India, Japan, South Korea, Mexico and Israel, collectively contributed 28% to the overall production of scientific publications and filed 18% of all patents. China made a 7% contribution to scientific publications and a 3% contribution to patent activities during this period (**Figure 1**).

Moving to the period from 2011 to 2021, China was the big story. China’s share of scientific publications jumped to 19% and the rest of the world rose to 30% while the United States and Europe both declined by 6% and 7%, respectively (**Figure 1**). Remarkably, China’s share of patents filed surged to 70% of all patents. This growth came at the expense of other nations, with the U.S. experiencing a 35% decline, Europe decreasing by 20%, and the rest of the world reporting a 12% reduction in patent activity (**Figure 1**) (For detailed patent and scientific publications distribution by country, please refer to **Tables A.1, A.2 in Appendix A**).

The increase in phenomics-related patents and publications between 2011 and 2021 can be attributed to several factors, including the resolution of previous issues related to affordability and access. Significant advancements and cost reductions over the past decade have made phenomics more accessible. Technological innovations, such as high-throughput phenotyping (HTP) platforms and the integration of AI and machine learning, have drastically reduced the time and cost of data collection and analysis (Mahlein, 2016). The development of open-source platforms and tools has further lowered costs by eliminating the need for expensive proprietary software (Tardieu et al., 2017). Collaborative efforts and consortia have facilitated the sharing of phenotyping facilities, reducing individual costs and enhancing research capabilities (Araus and Cairns, 2014).

Additionally, increased funding and support from governments and research institutions have helped establish and maintain phenotyping infrastructure, further mitigating initial high costs. Examples of such funding include the UK Biotechnology and Biological Sciences Research Council investing approximately $490 million (£388 million) between 2008 and 2019 to support plant science research, including significant investments in phenotyping infrastructure. This funding supported facilities such as the National Plant Phenomics Centre, the John Innes Centre, and Rothamsted Research, which are equipped with advanced phenotyping platforms (UK Research and Innovation, 2021). The European EMPHASIS project, funded under the EU Horizon 2020 program, invested approximately $8.4 million (€7 million) between 2015 and 2019 to develop a pan-European infrastructure for plant phenotyping (EMPHASIS, 2015). The French National Research Agency’s Investments for the Future Program (PIA), established in 2010, committed $50 billion (€47 billion) by 2020 to various research initiatives, including significant investments in plant phenomics (European Commission, 2017). Additionally, the National Science Foundation’s (NSF) Plant Genome Research Program (PGRP) has provided approximately $30 million annually to support plant genomics and phenomics research (NSF, 2020). These advancements have collectively improved the affordability and accessibility of phenomics, enabling broader engagement in phenotype studies.

From 2000 to 2010, the top five patent holders included Pioneer, U.S. (11 patents), Monsanto, U.S. (7 patents), BASF, Germany (5 patents), the Council of Scientific and Industrial Research, India (5 patents) and Cropdesign, Belgium (5 patents). Between 2011 and 2021, most significantly, all the top five patent holders were in China: University Nanjing (52 patents); Beijing Res Ct Information Tech Agriculture (30 patents); University Huazhong (27 patents); Nanjing Huitong Crop Phenotyping Research Institute Co Ltd (20 patents); and University Shandong (17 patents) (see **Table A.3 (a) in Appendix A** for more details).

In a broader perspective, China’s substantial growth in global patent filings for all fields has positioned it as the world’s top patent-filing country since 2011 (WIPO, 2022a).^5^ This achievement is supported by its adoption of international agreements like the Hague Agreement and the Marrakesh Treaty, reflecting its commitment to global Intellectual Property (IP) standards. Its consistent rise in the Global Innovation Index over the decade underscores its ongoing progress in fostering change. Additionally, China now hosts two of the world’s top five science and technology clusters, solidifying its status as a hub for pioneering research (WIPO, 2022b).^6^ By excelling in these areas and actively promoting IP protection and utilization, China has established itself as a vital player in global innovation. Some of the surge in patent filings is undoubtedly influenced by internal factors, including rapid economic growth and technological advancement, which create a conducive environment for innovation. The Chinese government’s emphasis on innovation and technology has catalyzed R&D activities, prompting both individuals and businesses to seek patent protection. Supportive policies and incentives, encompassing financial rewards, tax benefits, subsidies and streamlined patent application processes, have facilitated patent filings. These measures enable researchers to pursue patents, significantly boosting application numbers (He et al., 2018). China’s utility model and design patents have notably enhanced overall patenting, with lenient prerequisites and simplified procedures encouraging inventors and companies to secure protection for incremental innovations and product designs (Yu and Yip, 2021).^7^

Although China’s patent filings have surged, concerns persist regarding the quality and enforceability of these patents. According to Boeing and Mueller (2019), incentives focused on quantity may lead to more filings but lower-quality patents. The value of the underlying innovations varies. High-income economies prioritize programs that will deliver disruptive advancements that influence subsequent developments and increase citation rates. The challenge is that while citations are not flawless, they are the only readily available proxy for patent quality (WIPO, 2019). Comparing China and the U.S., the two top phenomics patent holders, significant differences in average citations emerge. Chinese patents average 3 citations (M=3.27; SD=3.75), while U.S. patents average about 14 citations (M=14.02; SD=37.44) from 2000 to 2011. In 2011-2021, the gap widened: Chinese patents still averaged 3 citations while U.S. patents were cited an average 19 times. This disparity impacts patent quality perception, innovation capabilities, competitiveness, IP strategies, and policy considerations. However, it is important to recognize the limitations of using citations as the sole measure of patent quality and impact.^8^

In the realm of scientific publications related to plant phenomics, from 2000 to 2010, leading organizations included: INRA (National Institute for Agricultural Research), France (50 publications); Max Planck Institute, Germany (35 publications); Cornell University, U.S. (28 publications); Wageningen University & Research, Netherlands (25 publications); and the University of California, Davis, U.S. (23 publications). Between 2011 and 2021, the top performers were USDA (U.S. Department of Agriculture) (119 publications); INRA, France (104 publications); Cornell University, U.S. (101 publications); China Agricultural University, China (94 publications); and Wageningen University & Research, Netherlands (93 publications) (see **Table A.3 (b) in Appendix A** for details).

Using publication citations as a measure of research quality and peer recognition, between 2000 and 2010, the U.S., UK, Germany, and the Netherlands outperformed other countries in securing highly-cited scientific publications. The following decade (2011-2021) saw China emerge as a significant contributor, while the U.S., Germany, and the UK continued to lead in terms of cited research. In the first decade, the U.S. was at the forefront with a total of 45,597 citations. In terms of average citations, the U.S. had an average citation score of 108.56, the UK 128.96, Germany and the Netherlands had averages about 125, with Portugal standing out due to its high average citation count of 397, although this was based on a relatively small total citation count.

From 2011 to 2021, the U.S. retained its position as the leader in total citations, reaching 81,023, despite a decrease in its average citation count to 53.41. China made significant progress, taking the second spot in total citations (TC=30,803) but with a lower average citation (M=31.15). Germany (TC=25,028; M=41.92), the UK (TC=17,257; M=44.82), and now Australia (TC=15,452; M=48.59) also demonstrated notable achievements in both total and average citation counts.

Throughout these two decades, the sustained dominance of the US in total citations was clear, even as the average citations per publication decreased. This trend is linked to an expanded research base with more publication. The significant rise of China in the second decade underscores its growing emphasis on digital agricultural innovation, reflecting a strategic redirection towards research areas with substantial impact^9^.

## International and sectoral shifts in collaborative efforts within plant phenomics

The public sector plays a crucial role in advancing plant phenomics research by providing funding and essential research infrastructure. This commitment is evident in the recent surge of investments in related facilities and programs. Examples include the EU EMPHASIS initiative, PHENOME in France, the Jülich Plant Phenotyping Centre in Germany, and the Australian Plant Phenomics Facility. Additionally, there has been a proliferation of networks, such as the International Plant Phenotyping Network, European Plant Phenotyping Network (EPPN), and the German Plant Phenotyping Network. The escalating demand for plant phenotyping has spurred the growth of a commercial sector. In 2018, this sector invested approximately US$159 million in plant phenotyping programs, with an anticipated annual growth rate of 11% through 2026 (Data Bridge Market Research, 2019).

Collaboration among plant phenomics researchers cultivates an environment that promotes the exchange of research findings, data and best practices across a diverse array of research institutions. This collaborative effort significantly elevates the overall quality and impact of plant phenomics research, promoting an ecosystem where expertise and knowledge flow relatively unrestrictedly, ultimately yielding superior solutions and outcomes. Of particular significance is the collaboration between the public and private sectors, which propels innovation and the creation of market-driven solutions. The private sector plays a crucial role by providing cutting-edge technology, valuable market insights, and essential funding, ensuring that research aligns with the agricultural industry’s needs and contributes to its long-term sustainability. This partnership enables large-scale experiments, knowledge dissemination, regulatory compliance, and the creation of lasting alliances, all critical for advancing plant phenomics research.

**Figure 2:**
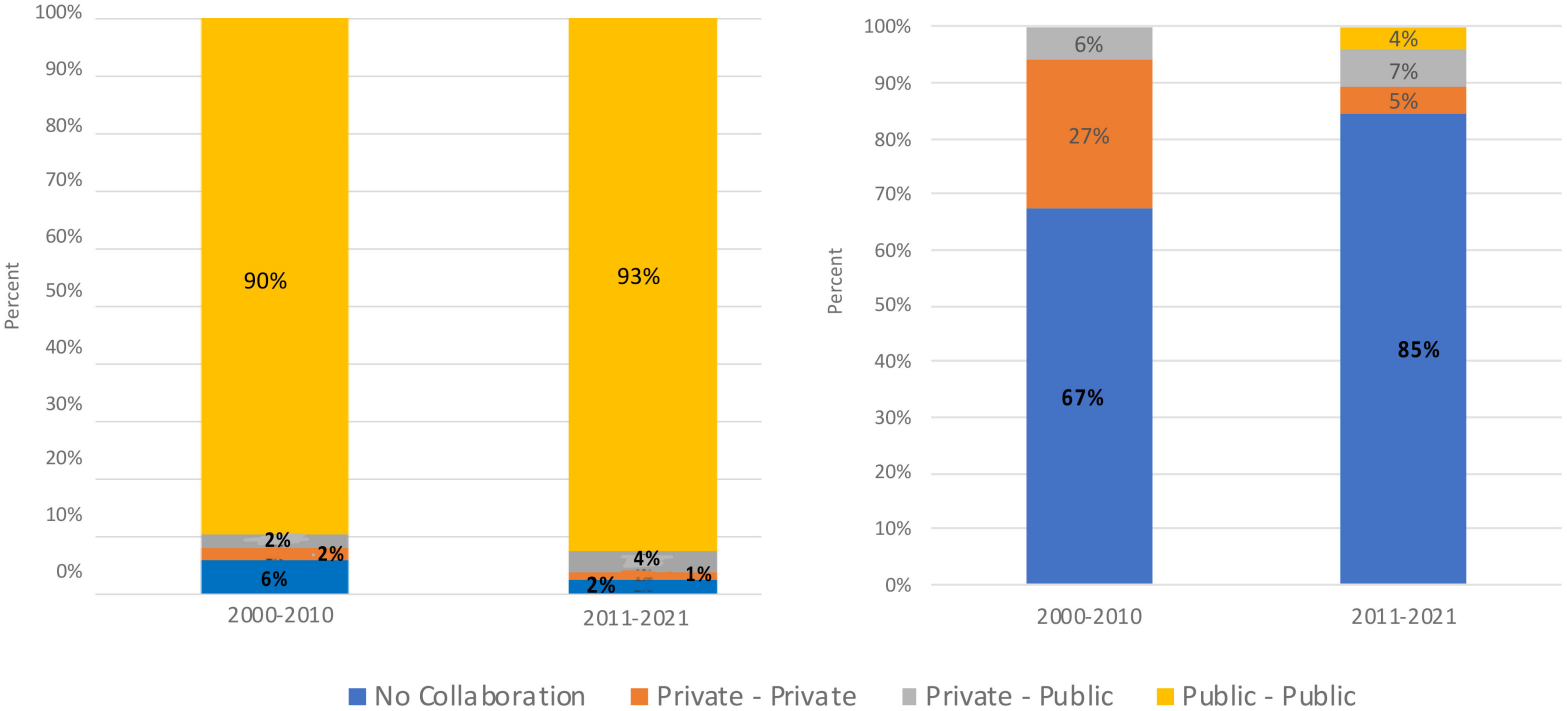
Trend in sectorial collaborations for scientific publications (left) and patents (right).

A review of phenomics scientific publication data from 2000 to 2021 shows a notable trend of collaboration, primarily featuring researchers associated with public institutions, and universities playing an important role in these collaborative efforts. Observed patterns suggest that universities have played a pivotal role in disseminating knowledge through collaborative research activities within the public sector. During this period, a relatively modest level of collaboration was observed between the public and private sectors, as well as within the private sector itself. These findings align with the typical trends observed in scientific publications, pointing towards the potential for greater cross-sectoral collaboration opportunities in future research activities (**Figure 2**, left) (see **Table A.4 (a), Appendix A** for details).

**Figure 2** (right) illustrates the evolving landscape of patent collaboration over the two decades. Between 2000 and 2010, private-private collaborations accounted for 27% of patents, while private-public collaborations made up 6%. Notably, there were no patents categorized under public-public collaboration during this decade. However, from 2011 to 2021, private-private collaborations decreased to 5%, while private-public collaborations increased to 7%. A small fraction of patents, approximately 4%, fell under the category of public-public collaboration (see **Table A.4 (b), Appendix A** for details).

To improve collaboration and bridge the gap between the private and public sectors, several measures can be taken. The private sector plays a critical role by developing high-throughput phenotyping platforms, integrating AI and machine learning for data analysis, and funding R&D activities. Understanding market demands and consumer preferences ensures that research efforts target traits beneficial to farmers and consumers. Enhancing collaboration can be achieved by promoting the development of scalable, accessible phenotyping technologies and better integrating them with breeding programs. Improving data management and sharing practices will facilitate the exchange of valuable insights between sectors. Additionally, providing enhanced training and capacity-building for researchers and breeders will ensure effective utilization of these technologies. By strengthening partnerships and leveraging the strengths of both sectors, plant phenomics research can more effectively align with the agricultural industry’s needs, contributing to its long-term sustainability. This approach will ensure that innovations in plant phenomics lead to practical applications that benefit the entire agricultural value chain.

**Figure 3:**
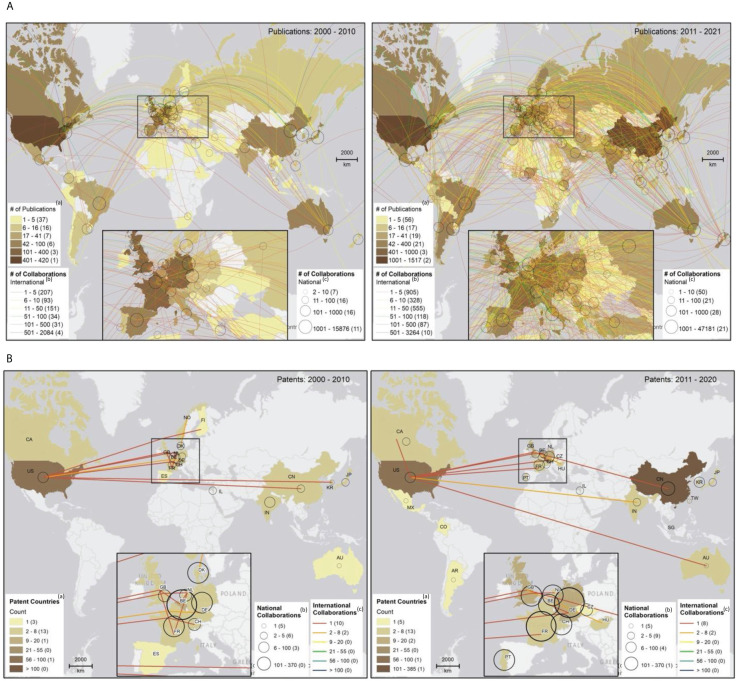
**(A)** National and international collaborations in scientific publications, 2000-2010 (left) and 2011-2021 (right). **^(a)^** Data in parentheses show countries in each publication count range;**^(b)^** data in parentheses indicate international collaboration instances within each range; and **^(c)^** data in parentheses signify national collaboration instances within each range. Country-specific data has been integrated into a worldwide spatial layer (https://esri.maps.arcgis.com/home/item.html?id=ac80670eb213440ea5899bbf92a04998#)! to visualize collaboration networks between countries based on their capital city locations. In ArcMap 10.8.1, we created the map layout and employed a manual classification scheme for mapping publications, national collaborations, and international collaborations. **(B)** National and international collaborations in patents, 2000-2010 (left) and 2011-2021 (right). **^(a)^** Data in parentheses show countries in each publication count range;**^(b)^** data in parentheses signify national collaboration instances within each range; and **^(c)^** data in parentheses indicate international collaboration instances within each range. Country-specific data has been integrated into a worldwide spatial layer (https://esri.maps.arcgis.com/home/item.html?id=ac80670eb213440ea5899bbf92a04998#) to visualize collaboration networks between countries. In ArcMap 10.8.1, we created the map layout and employed a manual classification scheme for mapping patents, national collaborations, and international collaborations.

**Figure 4:**
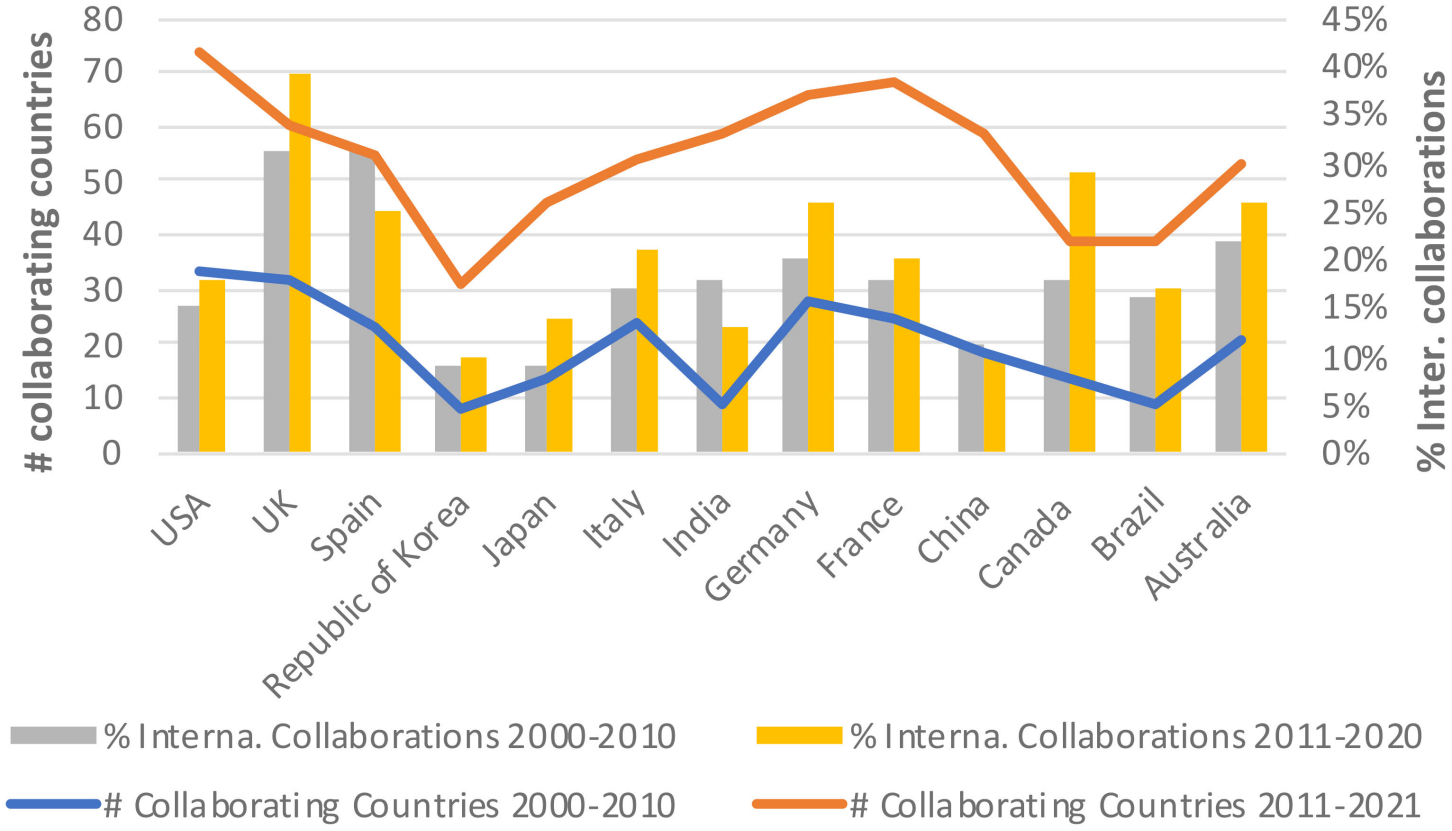
International collaborative efforts in scientific publishing.

**Figures 3A, B** map the evolution of international and national collaborations in plant phenomics over the two periods, highlighting partnerships between scientific authors (**Figure 3A**) and patent inventors (**Figure 3B**). We found that collaborations, both in scientific publications and patents, were largely concentrated within individual nations, especially among high-income economies (**Figures 3A, B**). However, a substantial increase in international collaboration was observed between 2011 and 2021, in contrast to the preceding decade. As illustrated in **Figure 4**, between 2000 and 2011 the proportion of publications involving international collaboration ranged from 9% to 31%, while the number of collaborating countries varied from 8 to 33. From 2011 to 2021, the percentage fluctuated between 10% and 39%, with the number of collaborative nations expanding from 31 to 74. During 2000 to 2010, the U.S. took the lead in international collaborations, working with 33 different countries. It was followed by the United Kingdom (32), Germany (28), France (25), Italy (24), and Australia (21). In 2011 to 2021, the U.S. continued to maintain its position at the forefront, collaborating with 74 countries. Germany (68), France (66), the United Kingdom (60), and China (59) also played significant roles in international collaborations. Notably, China’s international collaboration rate remained relatively stable at approximately 11% throughout the entire period, but it made substantial strides in expanding its collaborative network from 18 to 59 countries during the 2011-2021 period (**Figure 4**) (see **Table A.4 (b), Appendix A** for details).

With patents, we see significantly fewer cross-border collaborations engagements. This suggests a more localized approach to innovation and knowledge dissemination within specific nations (**Figure 3B**). There was actually a 7% decline in the number of international collaborations per patent during the 2011-2021 period compared to 2000-2010. Applicants from the Netherlands, Germany, Canada, UK, and the U.S. have engaged relatively more in patent production partnerships with applicants from other countries (see **Table A5 (b) in Appendix A** for details).

Our analysis revealed a strong positive correlation between international collaboration and research output (r(189) = 0.97, p < 0.001), such that countries with higher research output engage in international collaborations with a larger number of countries and do so more frequently. In the context of plant phenotyping innovation production, particularly efforts focused on large-scale data and standardized analysis, international collaboration holds paramount importance. It provides researchers access to diverse global environments, encompassing varying conditions, soils, and climates, thereby enhancing the accuracy of phenotypic analysis, and ensuring the global relevance of research findings. International partnerships facilitate the accumulation of extensive datasets, leading to more precise insights into plant phenotypes. Moreover, collaborative efforts drive data standardization, ensuring interoperability and the seamless integration and comparison of data from diverse sources, ultimately strengthening the reliability of research outcomes. Additionally, cross-border resource sharing, including specialized equipment and expertise, reduces costs and elevates data quality, benefiting all participants in these collaborative endeavors.

Much like the challenges encountered in other realms of innovation production, international collaboration in plant phenotyping, encompassing both patents and scientific publications, can encounter hurdles. These encompass the intricate web of divergent IP regulations, which introduce complexities regarding matters of ownership, rights, and royalties, especially within the framework of multi-institutional partnerships. Furthermore, the complex legal and regulatory terrain, characterized by varying patent application requirements in each country, can present significant barriers. Collaborative research endeavors that entail data sharing face additional impediments due to data privacy laws, ownership, and interoperability issues, which constrain the seamless exchange of data across borders. Additionally, the elaborate process of technology transfer between nations, fraught with complexities such as export controls, licensing, and adherence to local regulations, often results in delays that can impede progress in patent collaboration.

## Global collaboration networks: scientific co-authors and patent co-applicants

**Figure 5:**
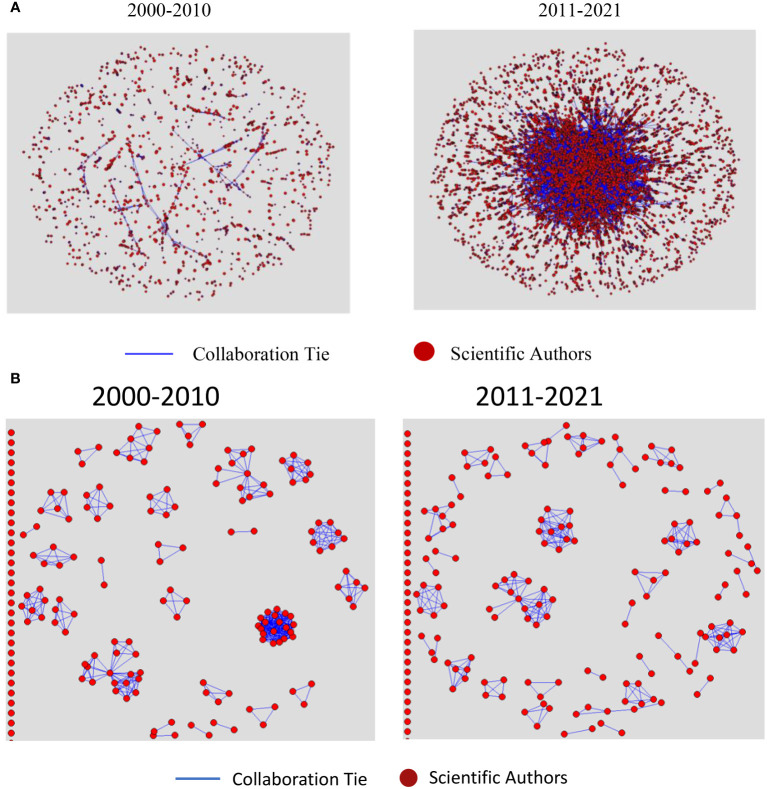
**(A)** Global Collaboration Network among Scientific Co-Authors in Plant phenotyping, 2000-2010 and 2011-2021. **(B)** Global Collaboration Network among Patent Co-Applicants in Plant phenotyping, 2000-2010 and 2011-2021.

In this section, we report on our social network analyses (SNA) used to characterize the evolution of global collaborative patterns among scientific co-authors and patent co-applicants in plant phenotyping over the two periods under review. **Figures 5A, B** display the collaboration networks of co-authors and co-applicants, respectively, over the two decades, while **Table 1** presents the analytical results for these networks.

**Table 1:** SNA results: collaboration networks cohesion metrics for scientific publications and patents.

Metric	Definition	Publications		Patents	
		2000-2010	2011-2021	2000-2010	2011-2021
Density	Proportion of actual collaborations to all possible collaborations.	0.0022	0.0008	0.03	0.00
Average Degree	The average number of collaborators an author has across all publications.	14.22	21.36	4.93	1.53
Average Distance	Average number of steps to connect two authors through collaborations.	2.54	6.96	1.27	1.25
Diameter	Maximum number of steps to connect any two authors in the network.	9	27	2.00	3.00
Fragmentation	Proportion of authors or groups that are isolated from the main collaboration network.	0.98	0.44	0.97	0.99
Closure	Proportion of realized connections to possible.	0.89	0.68	0.97	0.91

In scientific publication networks, **Figure 5A** illustrates a decline in network density during the second decade, decreasing from 0.0022 to 0.0008 as shown in **Table 1**. This decrease in cohesion can be attributed to the network’s considerable expansion – from 6,390 authors and 1,387 papers to 26,307 authors and 5,786 papers. Such an increase in a network’s size typically leads to a reduction in direct connections between authors, resulting in a broader yet less dense collaborative network. Despite this, the average degree centrality increased from 14.22 to 21.36, suggesting that, on average, authors engaged in more collaborations in the latter decade. This indicates a potential trend towards the formation of larger, more interdisciplinary, and extensive collaborative groups.

Significant changes were also noted in the average geodesic path length, which increased from 2.54 to 6.96, and the network diameter, which expanded from 9 to 27. The extended average distance, combined with the increased diameter, suggests that collaboration chains have become lengthier over time, possibly reflecting a diversification in research themes and a wider range of disciplines and geographical locations. In agricultural phenomics, research themes have diversified significantly, driven by enhanced interdisciplinary collaboration. Over the past two decades, the integration of fields such as plant biology, genetics, data science, agronomy, and ecology has driven substantial advancements (Fiorani and Schurr, 2013; Fahlgren et al., 2015; Tardieu et al., 2017; Araus et al., 2018; Yang et al., 2020). The World Intellectual Property Organization (WIPO, 2019) notes a shift from individual researchers to larger, specialized teams, evidenced by the rise in scientific publications authored by groups of six or more, reflecting the need for diverse expertise to address increasingly complex problems. Additionally, the geographical spread of collaboration has broadened. Empirical data show an increase in the average number of cities involved per research paper, from 5.24 in the first decade to 6.23 in the second decade (SD increased from 3.36 to 3.76). This expansion in collaboration distance and the growing network diameter suggest that research chains are becoming not only lengthier but also more diverse in disciplines and locations.

In addition, the network of co-authorship has become less fragmented over time, decreasing from 0.98 to 0.44, indicating a shift towards a more interconnected network. However, the concurrent increase in average distance and diameter suggests that this interconnectivity is characterized by more layered and complex collaboration patterns, transitioning from a centralized, compact network to a more decentralized, extensive one, involving longer collaboration paths with intermediaries. Even when accounting for the larger network size, the data indicate that, on average, authors were more distantly connected in terms of collaboration ties in the recent decade compared to the earlier period. Furthermore, a notable shift was observed in the network’s closure, which decreased from 0.89 in the first decade to 0.68 in the second, which suggests individual’s networks are more porous. In the earlier period, it was more likely for two collaborators of a given author to collaborate themselves, illustrating a closely-knit network. However, this became less prevalent in the second decade, with mutual collaborations among collaborators becoming less likely (**Table 1**).

In summary, over the second decade, despite a decrease in network density, there has been significant growth in the number of authors and papers within the scientific co-authorship network, indicating a substantial expansion of the research community and a trend towards broader and more intricate collaboration structures. This expansion, characterized by a higher average degree of collaboration per author, likely reflects a broadening scope of research interests and increased collaborative efforts, albeit within a more dispersed network structure. This evolution highlights a notable change in how research collaborations are formed and maintained in the field of plant phenomics, pointing towards larger but also more intricate and multi-level networks of co-authorship. These changes mirror an adaptation to the growing demands and complexities of scientific research, indicating a shift towards more diverse and interdisciplinary approaches to addressing scientific challenges.

The interdisciplinary approach in plant phenomics has evolved significantly beyond its initial focus on hardware and software engineering, incorporating diverse scientific disciplines to address complex plant biology questions. Researchers in plant biology and genetics provide insights into genetic and physiological mechanisms (Araus et al., 2018), while data scientists and bioinformaticians develop algorithms to analyze large-scale datasets (Yang et al., 2020). Agronomists and crop scientists optimize agricultural practices using phenomic tools (Tardieu et al., 2017), and ecologists study plant responses to environmental stressors (Fiorani and Schurr, 2013). Engineers continue to innovate with automated systems for field-based phenotyping (Houle et al., 2010). Recent advancements include integrating multi-omics data (Fahlgren et al., 2015), advanced imaging technologies, and machine learning algorithms to enhance phenotyping accuracy and efficiency (Yang et al., 2020). International collaborations, such as the International Plant Phenomics Network (IPPN), facilitate resource sharing and accelerate phenomic research (Araus et al., 2018). These developments underscore the critical role of interdisciplinary contributions in enhancing our understanding of plant biology and improving agricultural practices.

Facilities such as the Australian Plant Phenomics Facility (APPF) have been pioneering phenomics platforms since their establishment in 2009. Initially focused on developing advanced phenotyping technologies, these facilities have shifted towards optimizing and utilizing these technologies to generate valuable data and insights, particularly in plant breeding (Furbank et al., 2019; Australian Plant Phenomics Facility). This strategic shift reflects a broader trend within the field, prioritizing the practical application of innovations to address pressing agricultural challenges. The success of these efforts underscores the critical role of interdisciplinary collaboration, as integrating expertise from various scientific fields is essential for advancing plant phenomics and translating technological advancements into tangible agricultural benefits.

In patent networks, **Figure 5B** illustrates a decrease in network density throughout the second decade. Within these networks, applicants are connected if they have collaborated on patents. There is a significant variation in network sizes across the two periods, which impacts the ability to compare the two networks directly using various cohesion metrics. From 2000 to 2010, the first decade in the analysis, 189 applicants were actively involved in collaborations on patent production, whereas 360 were involved in the second decade, with the first period demonstrating a higher collaboration rate (78%) compared to the second (44%). **Table 1** indicates that the average degree of interactions fell sharply from the first decade to the second, dropping from 4.93 to 1.53. This signifies a decline in the frequency of collaborative efforts among patent applicants. Moreover, the interaction density decreased from 0.03 to nearly zero, indicating a reduction in network cohesiveness. The increase in network components from 68 to 245, along with the component ratio rising from 0.36 to 0.68, indicates growing fragmentation and the formation of isolated applicant clusters. The decline in network closure from 0.97 to 0.91, coupled with slight increases in both the average distance and the standard deviation of distances between applicants, also suggests a shift towards more dispersed interactions in the recent decade.

These results not only reveal structural changes in patent collaboration networks over time but also may have broader implications for the field’s capacity to innovate and conduct research. Reduced collaboration intensity and increased fragmentation could indicate a diversification in research directions within plant phenotyping. As the field broadens, applicants appear to be venturing into more specialized yet less interconnected research areas. This signals a shift towards more competitive innovation clusters, reflecting a strategic narrowing of focus among patent applicants, concentrating on specific areas of innovation within the plant phenotyping field.

The shift towards a more individualistic patenting could slow the diffusion of innovations, potentially extending the timeline for the development and application of new technologies. The increased dispersion of interactions and the rise in network components suggest difficulties for new entrants in integrating into existing collaboration networks, potentially impeding the flow of ideas and resources. This could also intensify competition for intellectual property rights, impacting the openness of research collaborations and the sharing of findings, with subsequent effects on the pace of innovation and accessibility of new technologies.

The evolution of collaboration networks in plant phenotyping reflects significant shifts in innovation dynamics, knowledge diffusion, and competitive strategies. Policies, especially with rising individualism and fragmentation, can play a key role, with international intellectual property rules critically shaping collaboration frameworks and guiding global knowledge and innovation exchange. Adjusting these policies might realign networks towards more integrated models, significantly enhancing innovation, knowledge sharing, and the effectiveness of the plant phenotyping research community.

## Leading global hubs for plant phenomics scientific publications

During the two decades, several cities have emerged as key contributors to the field of digital plant phenotyping, evidenced by their high publication outputs, concentration of researchers, collaborations and research facilities and institutions. These urban hubs have become important in driving the field forward, not only through the quantity of research they produce but also by attracting a significant number of specialists to their area. The presence of leading academic and research institutions within these cities further solidifies their role in shaping the direction and progress of agricultural phenotyping. Such cities stand out not only for their individual contributions but also for their roles within the broader collaborative network that spans the globe. Their influence extends beyond local boundaries, impacting the field on an international scale through extensive partnerships and joint ventures.

**Figure 6:**
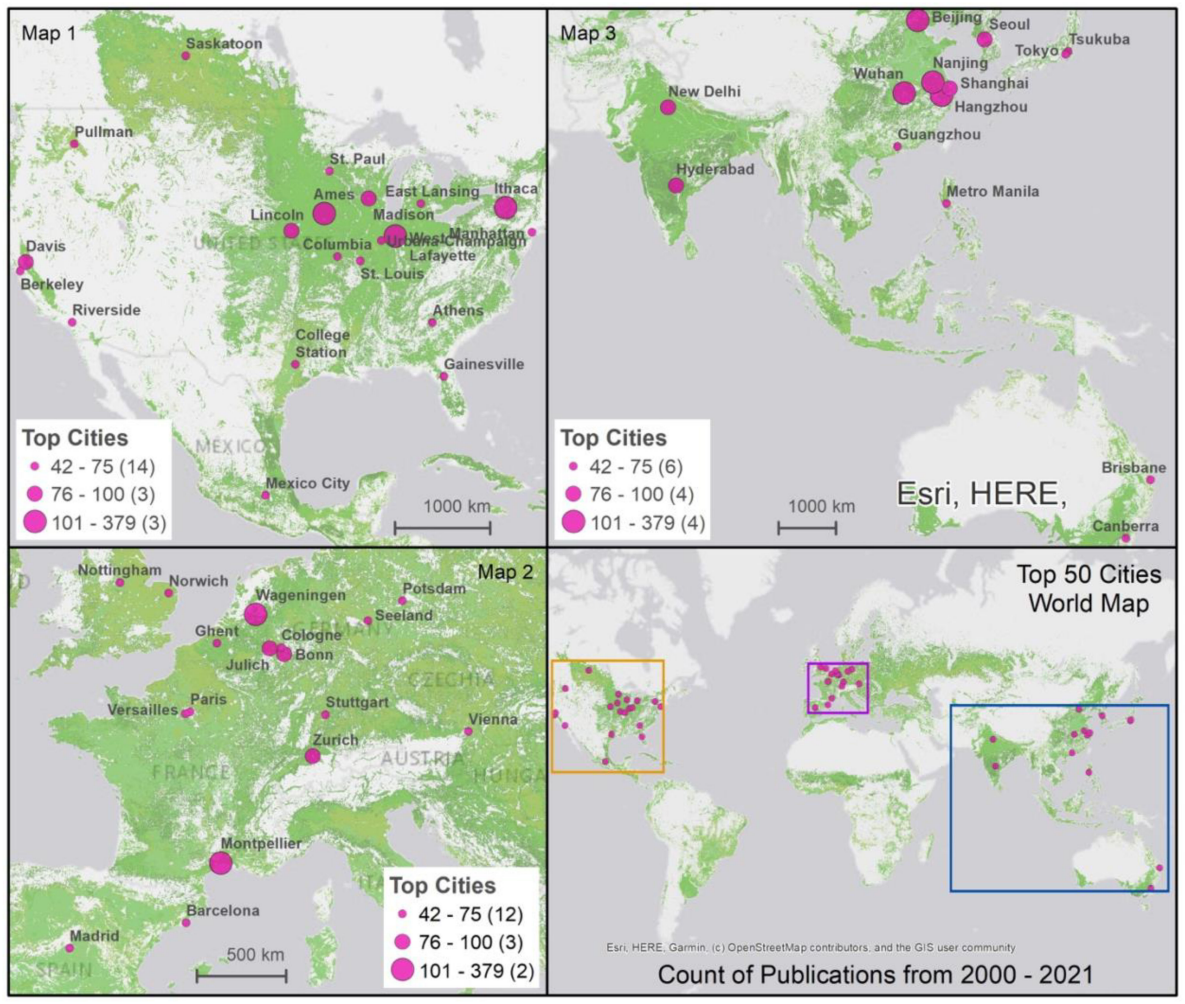
Leading Global Hubs for Plant Phenomics Research Scientific Publications and Cropland. Cropland layer is created using FAO open access data available at: https://www.fao.org/giews/earthobservation/asis/index_1.jsp?lang=en. The maps visualize key cities across the globe involved in plant phenomics research between 2000 and 2021. Each map uses differently sized pink circles to denote the volume of scientific publications, with larger circles representing more publications. The first three maps detail specific regions—North America, Europe, and Asia with Australia—highlighting major research hubs. The fourth map synthesizes the information, showing the global distribution of these hubs. The cropland areas are marked in light green, overlaying each map to indicate the geographical context of the research activity.

These hubs are highly geographically concentrated. The United States leads with 18 cities, followed by China and Germany with 6 each, France with 3, and Australia, India, Spain, the United Kingdom, and Japan, each with 2 cities. **Figure 6** shows that innovation hubs are strategically located either in major research centers or in areas crucial for agricultural production. For example, in the U.S. from 2000 to 2010, Ithaca (New York), Davis (California), Madison (Wisconsin), and West Lafayette (Indiana) were highly prolific centers, with research focused at the main universities in these locations: Cornell University, University of Wisconsin-Madison, and Purdue University, respectively. From 2011 to 2021, Cornell University in Ithaca and Purdue University in West Lafayette remained significant centers in the country. Two other locations, Ames (Iowa) and Lincoln (Nebraska), rose to prominence, with Iowa State University and the University of Nebraska respectively becoming prolific research centers. These hubs, known for their diverse agricultural outputs and as important centers for agricultural research, continue to be critical in advancing agricultural phenotyping and crop improvements, with most hosting dedicated research facilities and centers.

In Europe, Wageningen University, located in Wageningen, Netherlands, has consistently been a significant center of innovation in this field, ranking among the world’s most prolific institutions. Other notable European centers, including the Forschungszentrum in Jülich, Germany, the Max Planck Institute in Potsdam, Germany, and INRA (Institut National de la Recherche Agronomique) in Montpellier, France, also play crucial roles in advancing digital agricultural innovation research. These hubs, recognized for their varied agricultural outputs, are home to institutions with expertise spanning a broad array of agricultural domains.

In China, several prominent centers have emerged in the second decade, including China Agricultural University in Beijing, Huazhong Agricultural University in Wuhan, Nanjing Agricultural University in Nanjing, and Zhejiang University in Hangzhou. These institutions have been active in publishing agricultural research papers and have significantly contributed to the field. China’s innovation hubs extend from Beijing, a key research hub, to cities such as Wuhan, Nanjing, and Hangzhou, each located in areas renowned for agricultural production (e.g., rice farming).

**Figure 7:**
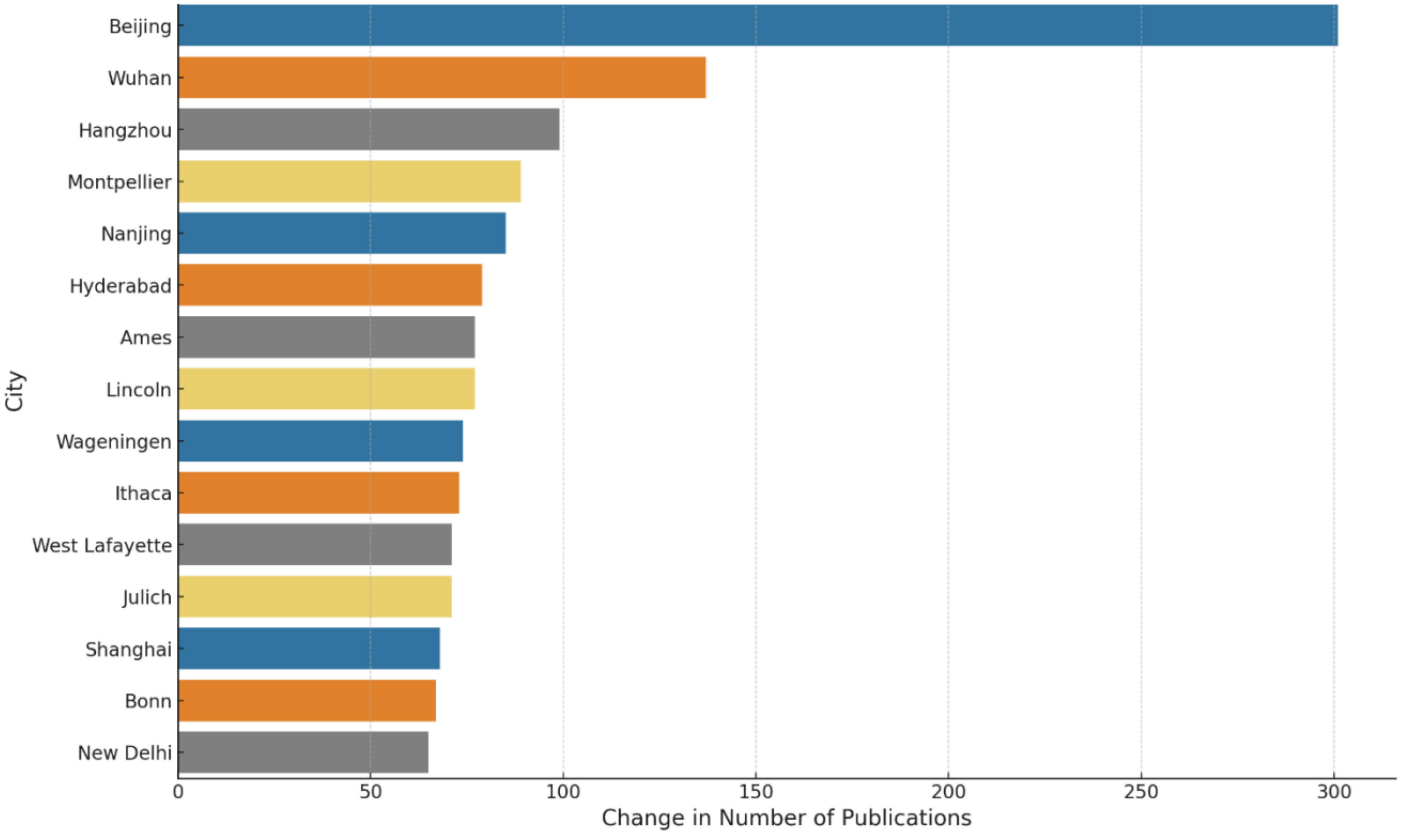
Top 15 cities by growth in plant phenotype scientific publications: a comparative analysis from 2000-2010 to 2011-2021.

Analysis reveals a significant positive correlation (0.61) between the publication outputs in the first decade (2000-2010) and the second decade (2011-2021), indicating that cities with higher publication counts in the first decade tend to sustain their productivity in the subsequent decade. **Figure 7** highlights the growth in scientific publications among the top 15 cities over these two decades, with Beijing, Wuhan, and Hangzhou in China experiencing the most notable increases.

Over the two decades, collaborations within and between these hubs have predominantly been intra-institutional, with a significant portion of research partnerships occurring among authors and researchers co-located at the same institutions, such as China Agricultural University in Beijing. When external collaborations do occur, they primarily involve other Chinese innovation hubs, like Huazhong Agricultural University in Wuhan, and occasionally international institutions, such as the University of California, Davis. This trend indicates a strong preference for local collaboration within these innovation centers, despite a gradual increase in the diversity and scope of their collaborative networks. Our findings demonstrate that while innovation hubs do engage in collaborations with other similar centers, the scope of these inter-center collaborations is limited compared to the more widespread intra-institutional partnerships.

As depicted in **Figure 6**, a significant characteristic of the leading 50 hubs is their deliberate proximity to agricultural zones, often affiliated with or situated around agricultural universities or institutions. These centers are profoundly engaged in plant phenotyping research, boasting extensive facilities and programs tailored for phenomics research in both field and controlled environments. **Table 2** presents the top 50 locations, detailing adjacent or onsite plant phenotyping facilities, with notable examples including the Plant Phenotyping Core Facility, Beijing, China; the Montpellier Plant Phenotyping Platforms in Montpellier, France; the Predictive Plant Phenomics Program at Ames, Iowa State University in the U.S.; and the Jülich Plant Phenotyping Center in Jülich, Germany. Other notable initiatives in developing economies include the CIMMYT phenotyping program in Mexico City, ICRISAT in Hyderabad, India, and IRRI near Metro Manila, Philippines. The establishment of plant phenotyping research facilities within the top 50 hubs, as detailed in **Table 2**, plays a pivotal role in advancing scientific research and driving regional economic growth. These centers become hubs for ecosystems that foster R&D in plant phenomics. The dynamic at play hinges on the fact that knowledge, especially in specialized areas like plant phenotyping, is less mobile than general information. It is rooted in specific locations through a combination of physical and intangible assets, including specialized equipment, unique environmental data, skilled personnel, and institutional knowledge. The strategic location of these hubs, near agricultural zones and associated with agricultural universities and institutions, enhances this effect. It creates an environment where research applications and theory enrich each other.

**Table 2:** Leading 50 cities in plant phenotype scientific publications and their associated or nearby plant phenotyping facilitiess.

City, Country	Plant Phenomics Facility/Program
Beijing, China	Plant Phenotyping Core Facility
Wuhan, China	Huazhong Agricultural University Crop Phenotyping Center
Montpellier, France	Montpellier Plant Phenotyping Platforms (PhenoArch, PhenoDyn and PhenoPsis)
Wageningen, Netherlands	The Netherlands Plant Eco-phenotyping Centre: NPEC; Wageningen University & Research
Hangzhou, China	Lab of Plant Phenomics and Safety & Quality Traits Improvement
Ithaca, USA	Phenomics - Innovation Lab for Crop Improvement, Cornell University
Ames, USA	Predictive Plant Phenomics Program, Iowa State University
Nanjing, China	Plant Phenomics Research Center, Nanjing Agricultural University
West Lafayette, USA	Phenotyping facility, Purdue University
Hyderabad, India	International Crops Research Institute for the Semi-Arid Tropics (ICRISAT)
Lincoln, USA	Plant Phenotyping - Agricultural Research Division
Julich, Germany	Jülich Plant Phenotyping Center (JPPC)
Davis, USA	High-Throughput In-Field Phenotyping Systems to Accelerate Breeding UC Davis
Madison, USA	High throughput plant phenotyping, Digital Agriculture Biological Systems Engineering, University of Wisconsin
New Delhi, India	ICAR-Indian Agricultural Research, Nanaji Deshmukh Plant Phenomics Centre
Bonn, Germany	PhenoRob, Universität Bonn
Zurich, Switzerland	Crop Phenotyping, ETH Zurich
Seoul, Republic of Korea	
Shanghai, China	
Seeland, Germany	IPK (Leibniz Institute of Plant Genetics and Crop Plant Research (IPK), Germany
College Station, USA	The Texas A&M Plant Growth and Phenotyping Facility
Canberra, Australia	The Australian Plant Phenomics Facility (APPF)
Colombia, USA	CIAT Phenotyping Platform
Madrid, Spain	Instituto Nacional de Investigación y Tecnología Agraria y Alimentaria (INIA)
St. Louis, USA	Donald Danforth Plant Science Center, The Bellwether Foundation Phenotyping Facility
Barcelona, Spain	University of Barcelona, Field Phenotype
Tsukuba, Japan	NARO Phenotyping Centre
Athens, USA	Phenomics & Plant Robotics Center University of Georgia Athens GA
Pullman, USA	Compact Plants Phenomics Center, WSU Plant phenomics, Washington State University
Manhattan, USA	
Norwich, UK	NRP Automated Crop Phenotyping Platform, John Innes Centre
Vienna, Austria	Vienna BioCenter Core Facilities, VBC, Vienna, Austria
Potsdam, Germany	Collaborative Research Centre 1644, Phenotypic plasticity in plants
Saskatoon, Canada	U of S Plant Phenotyping and Imaging Research Centre
Guangzhou, China	Shenzhen Branch, Guangdong Laboratory for Lingnan Modern Agriculture
East Lansing, USA	Center for Advanced Algal and Plant Phenotyping, Michigan State University
Ghent, Belgium	WIWAM Conveyor (PHENOVISION)
St. Paul, USA	Precision Agriculture Center, University of Minnesota
Mexico City, Mexico	CIMMYT, Breeder friendly phenotyping
Urbana-Champaign, USA	RIPE High-Throughput Phenotyping Facility, University of Illinois Urbana-Champaign
Cologne, Germany	
Gainesville, USA	Scanning Plant IoT (SPOT) Facility, Department of Agricultural & Biological Engineering at the University of Florida.
Stuttgart, Germany	
Berkeley, USA	Harmon Lab, College of Natural Resources University of California, Berkeley
Tokyo, Japan	Laboratory of Field Phenomics, University of Tokyo
Paris, France	The Phenomobile, ARVALIS
Brisbane, Australia	Predicting Phenotypes, ARC Centre of Excellence for Plant Success in Nature and Agriculture
Riverside, USA	JINKERSON LAB, University of California, Riverside
Metro Manila, Philippines	IRRI, Los Banos
Nottingham, UK	2D-RSAT, University of Nottingham
Versailles, France	INRA Île-De-France

## Discussion and future research

The period from 2010 to 2021 witnessed significant growth in plant phenomics research compared to the years 2000 to 2010, as evidenced by a noticeable increase in patents and scientific publications. This growth has been accompanied by fundamental shifts in the innovation landscape, affecting various geographic regions and sectors. Our analysis highlights the significant contributions from the U.S., Europe, and particularly China, with a special emphasis on China’s increasing dominance in patent filings. The substantial contributions from these countries reflect wide-ranging diversification of R&D efforts globally in this field, underscoring its growing international presence and crucial role in tackling complex agricultural challenges, including food security and climate change.

This analysis underscores the important role of interdisciplinary approaches and collaborations in driving innovations in plant phenomics. The growth of global research hubs and the emphasis on collaborative efforts illustrate the field’s movement towards more interconnected and diverse research networks. Within this context, the role of policy becomes evident. Policies that support international collaborations and pave the way for creating environments conducive to innovative research, promoting interdisciplinary projects, and facilitating international collaborations, including the creation of open-access databases and standardized protocols for data sharing, are crucial. These policies play a vital role in addressing challenges related to data management and integration, catalyzing innovation, and ensuring the equitable dissemination of technological advancements. The complexities of such partnerships, ranging from intellectual property issues to obstacles in data sharing, demand further consideration within the realm of agricultural phenomics. Identifying and examining successful models for international collaborations that effectively navigate these challenges can offer practical insights for fostering more efficient global partnerships in the future.

Future research directions could greatly benefit from expanding the measures from patents and publications to include a broader set of innovation indicators. While patents and publications provide important insights into the quantity and some quality aspects of research output, they do not fully capture the impact on technological advancement and agricultural productivity. To obtain a more nuanced understanding of the innovation ecosystem, incorporating diverse metrics such as R&D expenditure, the innovation-to-market conversion ratio, investment in scientific infrastructure, human capital development, and the effectiveness of knowledge transfer mechanisms is recommended. This approach can provide a more comprehensive view of the innovation ecosystem.

Analysis of the evolution of collaboration networks over time, through longitudinal network studies, could provide better understanding of the emergence of leaders. Coupled with an examination of the underlying mechanisms that determine or trigger these changes, this could shed light on ways of supporting the development of more effective and efficient networks of collaboration.

In addition to understanding changes over times in collaboration networks, studying the dynamics of research team productivity and innovation through SNA offers a promising pathway for future research in the field of agricultural phenomics. Research can be focused on patterns of collaborations within and across teams, providing insights into key individuals and relationships that facilitate knowledge flow and resource allocation, or on the optimal composition of research teams that enable an innovative environment. SNA also offers the potential to identify communication bottlenecks and workflow inefficiencies, that if addressed could enhance the efficiency of research processes. As interdisciplinary collaborations become increasingly vital to addressing complex challenges, understanding, and optimizing research team dynamics via SNA could significantly advance scientific innovation and productivity.

Incorporating spatial econometric models into future research could refine the identification of innovation clusters and the examination of how innovative activities are distributed spatially. This includes understanding the role of geographical proximity to research institutions and industries. Such models are also adept at evaluating the effects of policy decisions, infrastructure investments, and collaborative networks on innovation at both regional and broader levels.

The observed shift in patent filings towards China calls for a more nuanced analysis. Beyond mere volume, this trend points to underlying systemic influences like government policy, R&D investment, and innovation infrastructure. A critical examination of these factors is essential to better understand China’s emergence as a global leader in this field. The observed changes also raise the importance of evaluating and considering how different regions contribute to and share in the global knowledge economy of plant phenomics.

## Data availability statement

The raw data supporting the conclusions of this article will be made available by the authors, without undue reservation.

## Author contributions

LA: Writing – original draft, Writing – review & editing. PP: Writing – original draft, Writing – review & editing. AB: Writing – original draft, Writing – review & editing.

## Footnotes

1 Qualitative and quantitative traits (QTs) are the two genetic categories of traits. Qualitative traits are controlled by oligogenes, which produce a large effect on the trait phenotype, have high heritability, and provide discrete phenotypic variation. QTs, on the other hand, are governed by polygenes that have a small effect on the trait phenotype. Since the effects of all polygenes affecting a trait are cumulative, QTs display continuous phenotypic variation and cannot be classified into distinct phenotyping classes due to polygenic inheritance and environmental influences (Singh and Singh, 2015).

2 The low level of inheritance of polygenes and the influence of environmental factors that characterize QTs can result in deviation from the genetic variance of additive effects or breeding value, as there may be variance arising from dominant effects and epistatic effects that reduce heritability, as well as variance arising from the interaction between the genotype and environment (Awada et al., 2018).

3 Through genetic linkage mapping studies and genome-wide association studies (GWAS), researchers can identify candidate genes for various traits. These methods allow researchers to analyze a large number of genetic markers across the genome to identify regions that are associated with the trait of interest. These genes can then be further analyzed and characterized using functional genomics tools, such as CRISPR-Cas gene editing. Once the key genes are identified, they can be used in plant breeding programs to develop improved new crop varieties, using different methods, such as such as marker-assisted selection and genome editing (Thomson et al., 2022).

4 RGB imaging is a type of color imaging that captures red, green, and blue light wavelengths to create a full-color image. Chlorophyll fluorescence imaging measures the fluorescence emitted by chlorophyll molecules in response to light, which can provide insights into the photosynthetic efficiency of plants. Hyperspectral imaging captures light across a wider range of wavelengths than RGB imaging, which can help to identify subtle differences in plant physiology and stress. Thermal imaging captures the heat emitted by plants, which can be used to measure plant stress and water use efficiency. Finally, lidar imaging uses laser pulses to create a 3D map of the plant canopy, which can provide information on plant height, biomass, and architecture (Yang et al., 2020).

5 According to the WIPO (2022a), there were approximately 16.5 million patents in force worldwide. China accounted for 3.6 million of these patents, followed by the US with 3.3 million, Japan with 2 million, the Republic of Korea with 1.2 million and Germany with 877,7631. China saw the fastest growth in patents in force in 2021 with a growth rate of 17.6%, followed by Germany and the Republic of Korea with growth rates of 5.2% each.

6 East Asia is home to four out of the world’s five largest science and technology clusters. The Tokyo-Yokohama cluster holds the top position, followed by the Shenzhen-Hong Kong-Guangzhou cluster in South China and the Beijing cluster in North China. The fourth and fifth spots are occupied by a cluster in South Korea and another in the United States, respectively (WIPO, 2022c).

7 In China, utility model patents offer protection for minor or incremental inventions and feature streamlined procedures for patent filings. Similarly, design patents focus on safeguarding the aesthetic aspects of a product and benefit from comprehensive protection. The simplified procedures and reduced costs associated with obtaining utility model and design patents have encouraged companies and individuals to pursue patent protection, leading to a significant rise in patent filings in China (Yu and Yip, 2021).

8 The China National Intellectual Property Administration (CNIPA) is actively working on patent system reforms to enhance examination standards, improve patent quality, and align with international standards for genuine innovation and technological advancement (Yu and Yip, 2021). As part of their commitment to quality intellectual property services, CNIPA plans to terminate all patent subsidies by 2025 (The National Law Review, 2021).

9 Since our data allowed us to only analyze citation metrics, gaining a better understanding of the impact of phenomics discoveries on identifying traits and genes and their utilization in crop improvement necessitates analyzing their adoption by end users. Future studies should focus on collecting data through surveys, interviews, and case studies to measure adoption rates, application areas, and outcomes. Key factors include identifying technical, economic, and policy barriers and enablers. Quantitative and qualitative analyses are needed to assess the effectiveness and broader impact of phenomics technologies on agricultural innovation. This will provide a comprehensive evaluation of their impact.

## References

[B1] Anderson J. V. Wittenberg A. Li H. Berti M. T. (2019). High throughput phenotyping of Camelina sativa seeds for crude protein, total oil, and fatty acids profile by near infrared spectroscopy. Ind. Crops Prod. 137, 501–507. doi: 10.1016/j.indcrop.2019.04.075

[B2] Araus J. L. Cairns J. E. (2014). Field high-throughput phenotyping: the new crop breeding frontier. Trends Plant Sci. 19, 52–61. doi: 10.1016/j.tplants.2013.09.008 24139902

[B3] Araus J. L. Kefauver S. C. Zaman-Allah M. A. Olsen M. S. Cairns J. E. (2018). Translating high-throughput phenotyping into genetic gain. Trends Plant Sci. 23, 451–466. doi: 10.1016/j.tplants.2018.02.001 29555431 PMC5931794

[B4] Australian Plant Phenomics Facility (APPF) Australian Plant Phenomics Facility: Overview. Available online at: https://www.plantphenomics.org.au/about-us/.

[B5] Awada L. Phillips P. Smyth S. (2018). The adoption of automated phenotyping by plant breeders. Euphytica 214, 148. doi: 10.1007/s10681-018-2226-z

[B6] Awada L. Phillips P. W. B. Bogdan A. M. (2021). Governance and stewardship for research data and information sharing: Issues and prospective solutions in the transdisciplinary plant phenotyping and imaging research center network. Plants People Planet 4, 84–95. doi: 10.1002/ppp3.10238

[B7] Boeing P. Mueller E. (2019). Measuring China’s patent quality: Development and validation of ISR indices. China Econ. Rev. 57. doi: 10.1016/j.chieco.2019.101331

[B8] Data Bridge Market Research (2019). Global plant phenotyping market. Available online at: https://www.databridgemarketresearch.com/news/global-plant-phenotyping-market.

[B9] Dhondt S. Wuyts N. Inzé D. (2013). Cell to whole-plant phenotyping: the best is yet to come. Trends Plant Sci. 18, 428–439. doi: 10.1016/j.tplants.2013.04.008 23706697

[B10] Du J. Zhang Y. Guo X. Ma L. Shao M. Pan X.. (2016). Micron-scale phenotyping quantification and three-dimensional microstructure reconstruction of vascular bundles within maize stems based on micro-CT scanning. Funct. Plant Biol. 44, 10–22. doi: 10.1071/FP16117 32480542

[B11] EMPHASIS (2015). Bringing EMPHASIS to operation: European Infrastructure for multi-scale Plant Phenomics and Simulation for food security in a changing climate. Available online at: https://www.emphasisproject.eu.

[B12] Esri Maps (2023). World Countries represents the boundaries for the countries of the world. Available online at: https://esri.maps.arcgis.com/home/item.html?id=ac80670eb213440ea5899bbf92a04998.

[B13] European Commission (2017). Analysis of National Initiatives on Digitising European Industry France: Alliance Industries du Futur. Available online at: https://ec.europa.eu/futurium/en/system/files/ged/fr_country_analysis.pdf.

[B14] Fahlgren N. Gehan M. A. Baxter I. (2015). Lights, camera, action: high-throughput plant phenotyping is ready for a close-up. Curr. Opin. Plant Biol. 24, 93–99. doi: 10.1016/j.pbi.2015.02.006 25733069

[B15] Fiorani F. Schurr U. (2013). Future scenarios for plant phenotyping. Annu. Rev. Plant Biol. 64, 267–291. doi: 10.1146/annurev-arplant-050312-120137 23451789

[B16] Furbank R. T. Jimenez-Berni J. A. George-Jaeggli B. Potgieter A. B. Deery D. M. (2019). Field crop phenomics: enabling breeding for radiation use efficiency and yield potential. J. Exp. Bot. 70, 2165–2176. doi: 10.1111/nph.15817 30937909

[B17] He Z. L. Tong T. Zhang Y. He W. (2018). A database linking Chinese patents to China’s census firms. Sci. Data 5, 180042. doi: 10.1038/sdata.2018.42 29583142 PMC5956277

[B18] Houle D. Govindaraju D. R. Omholt S. (2010). Phenomics: the next challenge. Nat. Rev. Genet. 11, 855–866. doi: 10.1038/nrg2897 21085204

[B19] LeCun Y. Bengio Y. Hinton G. (2015). Deep learning. Nature 521, 436. doi: 10.1038/nature14539 26017442

[B20] Liu H. Bruning B. Garnett T. Berger B. (2020). Hyperspectral imaging and 3D technologies for plant phenotyping: from satellite to close-range sensing. Comput. Electron. Agric. 175, 105621. doi: 10.1016/j.compag.2020.105621

[B21] Ma Z. H. Mao Y. H. Gong L. Liu C. L. (2016). Smartphone-based visual measurement and portable instrumentation for crop seed phenotyping. IFAC-PapersOnLine 49, 259–264. doi: 10.1016/j.ifacol.2016.10.048

[B22] Mahlein A. K. (2016). Plant disease detection by imaging sensors – parallels and specific demands for precision agriculture and plant phenotyping. Plant Dis. 100, 241–251. doi: 10.1094/PDIS-03-15-0340-FE 30694129

[B23] Mochida K. Koda S. Inoue K. Hirayama T. Tanaka S. Nishii R.. (2019). Computer vision-based phenotyping for improvement of plant productivity: a machine learning perspective. GigaScience 8, giy153. doi: 10.1093/gigascience/giy153 30520975 PMC6312910

[B24] Mohanty S. P. Hughes D. P. Salathé M. (2016). Using deep learning for image-based plant disease detection. Front. Plant Sci. 7. doi: 10.3389/fpls.2016.01419 PMC5032846 27713752

[B25] Nakaya A. Isobe S. N. (2012). Will genomic selection be a practical method for plant breeding? Ann. Bot. 110, 1303–1316. doi: 10.1093/aob/mcs109 22645117 PMC3478044

[B26] National Science Foundation (NSF) (Plant Genome Research Program (PGRP). Available online at: https://new.nsf.gov/funding/opportunities/plant-genome-research-program-pgrp.

[B27] Singh B. D. Singh A. K. (2015). Marker-Assisted Plant Breeding: Principles and Practices (New Delhi: Springer). doi: 10.1007/978-81-322-2316-0

[B28] Song P. Wang J. Guo X. Yang W. Zhao C. (2021). High-throughput phenotyping: Breaking through the bottleneck in future crop breeding. Crop J. 3, 633–645. doi: 10.1016/j.cj.2021.03.015

[B29] Tardieu F. Cabrera-Bosquet L. Pridmore T. Bennett M. (2017). Plant phenomics, from sensors to knowledge. Curr. Biol. 27, R770–R783. doi: 10.1016/j.cub.2017.05.055 28787611

[B30] The National Law Review (2021). China to Cancel All Patent Subsidies, China IP Law Update. Available online at: https://natlawreview.com/article/china-to-cancel-all-patent-subsidies.

[B31] Thomas D. (2010). Gene-environment-wide association studies: emerging approaches. Nat. Rev. Genet. 11, 259–272. doi: 10.1038/nrg2764 20212493 PMC2891422

[B32] Thomson M. J. Biswas S. Tsakirpaloglou N. Septiningsih E. M. (2022). Functional allele validation by gene editing to leverage the wealth of genetic resources for crop improvement. Int. J. Mol. Sci. 23, 6565. doi: 10.3390/ijms23126565 35743007 PMC9223900

[B33] UK Research and Innovation (2021). BBSRC strategic delivery plan 2022 to 2025. Available online at: https://www.ukri.org/publications/bbsrc-strategic-delivery-plan/bbsrc-strategic-delivery-plan-2022-to-2025/.

[B34] WIPO (2019). World Intellectual Property Report 2019: The geography of innovation: Local hotspots, global networks. (Geneva: World Intellectual Property Organization). Available at: https://www.wipo.int/edocs/pubdocs/en/wipo_pub_944_2019.pdf.

[B35] WIPO (2022a). Worldwide IP Filings Reached New All-Time Highs in 2021, Asia Drives Growth (Geneva: WIPO Media Center). Available at: https://www.wipo.int/pressroom/en/articles/2022/article_0013.html.

[B36] WIPO (2022b). World IP Indicators: China Witnesses Substantial Growth Across the Board (AFD China Intellectual Property Law Office). Available at: file:///Users/lanaawada/Documents/SNA%20Phease%20II%20Internation%20/articles/World%20IP%20Indicators:%20China%20Witnesses%20Substantial%20Growth%20Across%20the%20Board%20-%20Lexology.html.

[B37] WIPO (2022c). Global Innovation Index 2022. What is the future of innovation-driven growth?, Global Innovation Index 2022: What is the future of innovation-driven growth? (wipo.int).

[B38] Xiao Q. Bai X. Zhang C. He Y. (2022). Advanced high-throughput plant phenotyping techniques for genome-wide association studies: a review. J. Adv. Res. 35, 215–230. doi: 10.1016/j.jare.2021.05.002 35003802 PMC8721248

[B39] Yang W. Feng H. Zhang X. Zhang J. Doonan J. H. Batchelor W. D.. (2020). Crop phenomics and high-throughput phenotyping: past decades, current challenges, and future perspectives. Mol. Plant 13, 187–214. doi: 10.1016/j.molp.2020.01.008 31981735

[B40] Yu R. Yip K. (2021). New Changes, New Possibilities: China’s Latest Patent Law Amendments Vol. 70 (GRUR International, Journal of European and international IP Law), 486–489. doi: 10.1093/grurint/ikaa201

[B41] Zhang C. Y. Si Y. S. Lamkey J. Boydston R. A. Garland-Campbell K. A. Sankaran S. (2018). High-throughput phenotyping of seed/seedling evaluation using digital image analysis. Agronomy 8, 63. doi: 10.3390/agronomy8050063

[B42] Zhang Y. Zhang N. (2018). Imaging technologies for plant high-throughput phenotyping: a review. Front. Agric. Sci. Eng. 5, 406–419. doi: 10.15302/J-FASE-2018242

[B43] Zhao C. Zhang Y. Du J. Guo X. Wen W. Gu S.. (2019). Crop phenomics: Current status and perspectives. Front. Plant Sci. 10. doi: 10.3389/fpls.2019.00714 PMC6557228 31214228

