# Supplementary material for: The evolution of plant phenomics: global insights, trends, and collaborations (2000-2021)
Source: Front Plant Sci. 2024 Jul 22;15:1410738. doi: 10.3389/fpls.2024.1410738 (PMC11298374; doi:10.3389/fpls.2024.1410738)
Supplement: Supplementary file 1 [file DataSheet_1.docx]

**Appendix A**

**Table A.1 The distribution of digital phenomics publications by countries**

| Country | 2000-2010 Number of Publications | 2011-2021 Number of Publications | Total Number of Publications | %  2000-2010 | %  2011-2021 |
| --- | --- | --- | --- | --- | --- |
| U.S. | 420 | 1517 | 1937 | 25% | 19% |
| China | 92 | 989 | 1081 | 7% | 19% |
| Germany | 139 | 597 | 736 | 10% | 10% |
| France | 120 | 404 | 524 | 9% | 7% |
| UK | 112 | 385 | 497 | 8% | 7% |
| India | 26 | 325 | 351 | 2% | 6% |
| Australia | 73 | 318 | 391 | 5% | 5% |
| Spain | 56 | 293 | 349 | 4% | 5% |
| Japan | 94 | 250 | 344 | 7% | 4% |
| Italy | 41 | 229 | 270 | 3% | 4% |
| Brazil | 23 | 180 | 203 | 2% | 3% |
| Canada | 61 | 179 | 240 | 4% | 3% |
| Republic of Korea | 30 | 155 | 185 | 2% | 3% |
| Netherlands | 50 | 132 | 182 | 4% | 2% |
| Belgium | 36 | 122 | 158 | 3% | 2% |
| Switzerland | 30 | 117 | 147 | 2% | 2% |
| Mexico | 24 | 107 | 131 | 2% | 2% |
| Austria | 7 | 76 | 83 | 1% | 1% |
| Denmark | 14 | 72 | 86 | 1% | 1% |
| Czech Republic | 8 | 68 | 76 | 1% | 1% |
| Sweden | 16 | 61 | 77 | 1% | 1% |
| Israel | 15 | 61 | 76 | 1% | 1% |
| Philippines | 14 | 55 | 69 | 1% | 1% |
| Argentina | 14 | 54 | 68 | 1% | 1% |
| Poland | 8 | 52 | 60 | 1% | 1% |
| New Zealand | 7 | 45 | 52 | 1% | 1% |
| Scotland | 16 | 43 | 59 | 1% | 1% |
| Portugal | 7 | 40 | 47 | 1% | 1% |
| Pakistan | 1 | 40 | 41 | 0% | 1% |
| Taiwan | 4 | 39 | 43 | 0% | 1% |
| Iran | 4 | 38 | 42 | 0% | 1% |
| Saudi Arabia | 2 | 37 | 39 | 0% | 1% |
| Russia | 6 | 35 | 41 | 0% | 1% |
| Chile | 2 | 35 | 37 | 0% | 1% |
| South Africa | 4 | 34 | 38 | 0% | 1% |
| Greece | 1 | 33 | 34 | 0% | 1% |
| Colombia | 6 | 31 | 37 | 0% | 1% |
| Finland | 5 | 31 | 36 | 0% | 1% |
| Kenya | 0 | 31 | 31 | 0% | 1% |
| Ireland | 1 | 30 | 31 | 0% | 1% |
| Malaysia | 0 | 27 | 27 | 0% | 0% |
| Norway | 7 | 24 | 31 | 1% | 0% |
| Hungary | 6 | 19 | 25 | 0% | 0% |
| Turkey | 3 | 19 | 22 | 0% | 0% |
| Nigeria | 0 | 19 | 19 | 0% | 0% |
| Singapore | 7 | 17 | 24 | 1% | 0% |
| Serbia | 0 | 17 | 17 | 0% | 0% |
| Zimbabwe | 0 | 16 | 16 | 0% | 0% |
| Thailand | 6 | 15 | 21 | 0% | 0% |
| Egypt | 2 | 15 | 17 | 0% | 0% |
| Bangladesh | 0 | 13 | 13 | 0% | 0% |
| Morocco | 2 | 12 | 14 | 0% | 0% |
| Peru | 2 | 12 | 14 | 0% | 0% |
| Ethiopia | 1 | 12 | 13 | 0% | 0% |
| Tunisia | 1 | 12 | 13 | 0% | 0% |
| Slovenia | 2 | 9 | 11 | 0% | 0% |
| Indonesia | 1 | 9 | 10 | 0% | 0% |
| Slovakia | 1 | 9 | 10 | 0% | 0% |
| Vietnam | 1 | 8 | 9 | 0% | 0% |
| Bulgaria | 1 | 7 | 8 | 0% | 0% |
| Côte d’Ivoire | 0 | 7 | 7 | 0% | 0% |
| Oman | 1 | 6 | 7 | 0% | 0% |
| Hong Kong | 0 | 6 | 6 | 0% | 0% |
| Nepal | 0 | 6 | 6 | 0% | 0% |
| Uganda | 0 | 6 | 6 | 0% | 0% |
| Croatia | 3 | 5 | 8 | 0% | 0% |
| Estonia | 2 | 5 | 7 | 0% | 0% |
| Niger | 1 | 5 | 6 | 0% | 0% |
| Cuba | 0 | 5 | 5 | 0% | 0% |
| Cyprus | 0 | 5 | 5 | 0% | 0% |
| Mali | 1 | 4 | 5 | 0% | 0% |
| Ukraine | 1 | 4 | 5 | 0% | 0% |
| Ecuador | 0 | 4 | 4 | 0% | 0% |
| Kazakhstan | 0 | 4 | 4 | 0% | 0% |
| Lithuania | 0 | 4 | 4 | 0% | 0% |
| Romania | 0 | 4 | 4 | 0% | 0% |
| Uruguay | 2 | 3 | 5 | 0% | 0% |
| Ghana | 1 | 3 | 4 | 0% | 0% |
| Belarus | 0 | 3 | 3 | 0% | 0% |
| Benin | 0 | 3 | 3 | 0% | 0% |
| Iraq | 0 | 3 | 3 | 0% | 0% |
| Jordan | 0 | 3 | 3 | 0% | 0% |
| Senegal | 0 | 3 | 3 | 0% | 0% |
| Tanzania | 0 | 3 | 3 | 0% | 0% |
| Luxembourg | 2 | 2 | 4 | 0% | 0% |
| Algeria | 1 | 2 | 3 | 0% | 0% |
| Lao People’s Democratic Republic | 1 | 2 | 3 | 0% | 0% |
| Lebanon | 1 | 2 | 3 | 0% | 0% |
| Sri Lanka | 1 | 2 | 3 | 0% | 0% |
| Armenia | 0 | 2 | 2 | 0% | 0% |
| Azerbaijan | 0 | 2 | 2 | 0% | 0% |
| Democratic Republic of the Congo | 0 | 2 | 2 | 0% | 0% |
| Dominican Republic | 0 | 2 | 2 | 0% | 0% |
| Latvia | 0 | 2 | 2 | 0% | 0% |
| Malawi | 0 | 2 | 2 | 0% | 0% |
| Mauritius | 0 | 2 | 2 | 0% | 0% |
| UAE | 0 | 2 | 2 | 0% | 0% |
| Zambia | 0 | 2 | 2 | 0% | 0% |
| Syria | 4 | 1 | 5 | 0% | 0% |
| Cameroon | 1 | 1 | 2 | 0% | 0% |
| Costa Rica | 1 | 1 | 2 | 0% | 0% |
| Afghanistan | 0 | 1 | 1 | 0% | 0% |
| Angola | 0 | 1 | 1 | 0% | 0% |
| Bolivia | 0 | 1 | 1 | 0% | 0% |
| Burkina Faso | 0 | 1 | 1 | 0% | 0% |
| Democratic People’s Republic of Korea | 0 | 1 | 1 | 0% | 0% |
| Djibouti | 0 | 1 | 1 | 0% | 0% |
| French Guiana | 0 | 1 | 1 | 0% | 0% |
| Georgia | 0 | 1 | 1 | 0% | 0% |
| Guatemala | 0 | 1 | 1 | 0% | 0% |
| Honduras | 0 | 1 | 1 | 0% | 0% |
| Madagascar | 0 | 1 | 1 | 0% | 0% |
| Moldova | 0 | 1 | 1 | 0% | 0% |
| Myanmar | 0 | 1 | 1 | 0% | 0% |
| Nicaragua | 0 | 1 | 1 | 0% | 0% |
| Papua New Guinea | 0 | 1 | 1 | 0% | 0% |
| Paraguay | 0 | 1 | 1 | 0% | 0% |
| Republic of Panama | 0 | 1 | 1 | 0% | 0% |
| Saint Kitts and Nevis | 0 | 1 | 1 | 0% | 0% |
| Sudan | 0 | 1 | 1 | 0% | 0% |
| Togo | 0 | 1 | 1 | 0% | 0% |
| Albania | 1 | 0 | 1 | 0% | 0% |
| Venezuela | 1 | 0 | 1 | 0% | 0% |

**Table A.2 The distribution of digital phenomics patents by countries**

| Country | 2000-2010  Patent number | 2011-2021  Patent number | Total  Patent number | 2000-2010 % | 2011-2021% |
| --- | --- | --- | --- | --- | --- |
| China | 3 | 381 | 384 | 3% | 70% |
| United States | 54 | 94 | 148 | 52% | 17% |
| Germany | 7 | 11 | 18 | 7% | 2% |
| United Kingdom | 2 | 8 | 10 | 2% | 1% |
| India | 7 | 3 | 10 | 7% | 1% |
| Canada | 2 | 6 | 8 | 2% | 1% |
| Japan | 5 | 3 | 8 | 5% | 1% |
| South Korea | 1 | 7 | 8 | 1% | 1% |
| Netherlands | 4 | 4 | 8 | 4% | 1% |
| Belgium | 7 | 0 | 7 | 7% | 0% |
| Switzerland | 1 | 6 | 7 | 1% | 1% |
| France | 1 | 6 | 7 | 1% | 1% |
| Israel | 3 | 4 | 7 | 3% | 1% |
| Taiwan | 0 | 4 | 4 | 0% | 1% |
| Australia | 1 | 2 | 3 | 1% | 0% |
| Denmark | 2 | 0 | 2 | 2% | 0% |
| Spain | 1 | 1 | 2 | 1% | 0% |
| Norway | 2 | 0 | 2 | 2% | 0% |
| Portugal | 0 | 2 | 2 | 0% | 0% |
| Argentina | 0 | 1 | 1 | 0% | 0% |
| Colombia | 0 | 1 | 1 | 0% | 0% |
| Czech Republic | 0 | 1 | 1 | 0% | 0% |
| Finland | 1 | 0 | 1 | 1% | 0% |
| Mexico | 0 | 1 | 1 | 0% | 0% |
| Singapore | 0 | 1 | 1 | 0% | 0% |

**Table A.3(a). Leading countries in phenomics patent holders**

| Applicant Name | Country | Sector | 2000-2010 Patent Count | 2011-2021 Patent Count | Total Patents |
| --- | --- | --- | --- | --- | --- |
| Univ Nanjing | China | University | 0 | 52 | 52 |
| Beijing Res Ct Information Tech Agriculture | China | Public Org | 0 | 30 | 30 |
| Univ Huazhong | China | University | 0 | 27 | 27 |
| Nanjing Huitong Crop Phenotyping Res Institute Co Ltd | China | Private Org | 0 | 20 | 20 |
| Pioneer Hi Bred Int | U.S. | Private Org | 11 | 9 | 20 |
| Univ Shandong | China | University | 0 | 17 | 17 |
| Univ Zhejiang | China | University | 0 | 12 | 12 |
| Univ Beijing | China | University | 0 | 11 | 11 |
| Monsanto Technology Llc | U.S. | Private Org | 7 | 9 | 16 |
| Shanghai Agripheno Agricultural Tech Co Ltd | China | Private Org | 0 | 9 | 9 |
| Basf | Germany | Private Org | 5 | 6 | 11 |
| Univ Henan | China | University | 0 | 8 | 8 |
| Hunan Hybrid Rice Res Ct | China | Public Org | 0 | 7 | 7 |
| Univ Fujian Agriculture & Forestry | China | University | 0 | 6 | 6 |
| Univ South China | China | University | 0 | 6 | 6 |
| Council Scient Ind Res | India | Government | 5 | 0 | 5 |
| Cropdesign Nv | Belgium | Private Org | 5 | 0 | 5 |
| Inst Botany Cas | China | Public Org | 0 | 5 | 5 |
| Jiangsu Acad Agricultural Sci | China | University | 0 | 5 | 5 |
| Northeast Inst Geo & Agroecolo | China | Public Org | 0 | 5 | 5 |
| Univ Northwest A&F | China | University | 0 | 5 | 5 |
| Univ Shanghai Jiaotong | China | University | 0 | 5 | 5 |
| Pfizer | U.S. | Private Org | 3 | 0 | 3 |
| Dekalb Genetics Corp | U.S. | Private Org | 2 | 0 | 2 |
| Fluidigm Corp | U.S. | Private Org | 2 | 0 | 2 |
| Karchi Hagai | Israel | Individual | 2 | 0 | 2 |
| Keygene Nv | Netherlands | Private Org | 2 | 0 | 2 |
| Meissner Rafael | Israel | Individual | 2 | 0 | 2 |
| Mironov Vladimir | Belgium | Individual | 2 | 0 | 2 |
| Landbrukshoegskole Inst | Norway | Public Org | 2 | 0 | 2 |
| Ronen Gil | Israel | Individual | 2 | 0 | 2 |
| Rosetta Inpharmatics Llc | U.S. | Private Org | 2 | 0 | 2 |
| Samuel Roberts Noble Inc | U.S. | Private Org | 2 | 0 | 2 |
| Voon Gerard | Canada | Individual | 2 | 0 | 2 |
| Abadie Tabare E | U.S. | Individual | 1 | 0 | 1 |
| Agrigenetics Inc | U.S. | Private Org | 1 | 0 | 1 |
| Akai Tatsuo | Japan | Individual | 1 | 0 | 1 |

**Table A.3(b). Leading countries in Phenomics Scientific Publications**

| Author Affiliation | Country | Organization Sector | 2000-2010 Publication Count | 2011-2021 Publication Count | Total Publications |
| --- | --- | --- | --- | --- | --- |
| USDA | U.S. | Government | 19 | 119 | 138 |
| INRA | France | Public Org | 50 | 104 | 154 |
| Cornell University | U.S. | University | 28 | 101 | 129 |
| Wageningen University & Research | Netherlands | University | 25 | 93 | 118 |
| Max Planck Institute | Germany | Public Org | 35 | 82 | 117 |
| Iowa State University | U.S. | University | 15 | 91 | 106 |
| Purdue University West Lafayette | U.S. | University | 18 | 88 | 106 |
| China Agricultural University | China | University | 0 | 94 | 94 |
| CSIRO | Australia | Government | 21 | 73 | 94 |
| Forschungszentrum Jülich | Germany | Public Org | 15 | 82 | 97 |
| University of Nebraska, Lincoln | U.S. | University | 0 | 84 | 84 |
| Huazhong Agricultural University | China | University | 0 | 83 | 83 |
| Institute of Plant Genetics and Crop Plant Research | Germany | Public Org | 22 | 68 | 90 |
| University of California, Davis | U.S. | University | 23 | 0 | 23 |
| University of Wisconsin, Madison | U.S. | University | 21 | 0 | 21 |
| John Innes Centre | UK | Public Org | 20 | 0 | 20 |
| University of Tokyo | Japan | University | 19 | 0 | 19 |
| RIKEN | Japan | Public Org | 17 | 0 | 17 |
| University of Minnesota | U.S. | University | 17 | 0 | 17 |
| Agriculture and Agri-Food Canada | Canada | Government | 16 | 0 | 16 |
| Ghent University | Belgium | University | 15 | 0 | 15 |
| University of Florida | U.S. | University | 15 | 0 | 15 |
| International Rice Research Institute | Philippines | Public Org | 13 | 0 | 13 |
| International Maize and Wheat Improvement Center | Mexico | Public Org | 0 | 75 | 75 |
| University of Bonn | Germany | University | 0 | 70 | 70 |
| Nanjing Agricultural University | China | University | 0 | 68 | 68 |
| Zhejiang University | China | University | 0 | 68 | 68 |
| International Crops Research Institute for the Semi-Arid Tropics | India | Public Org | 0 | 67 | 67 |
| University of Nottingham | UK | University | 0 | 66 | 66 |
| Texas A&M University | U.S. | University | 0 | 65 | 65 |

**Table A.4(a) Sectorial collaborations for scientific publications**

| Entity / Period | Public Org. | | | | Private Org. | | | | | | No-collaboration | | | | |
| --- | --- | --- | --- | --- | --- | --- | --- | --- | --- | --- | --- | --- | --- | --- | --- |
|  | **2000-2010** | | **2011-2021** | **2000-2010** | | | | **2011-2021** | | | | **2000-2010** | | **2011-2021** |  |
| Public org. | 90% | | 93% | 2% | | | | 4% | | | | 6% | | 2% |  |
| Private org. | 2% | | 4% | 2% | | | | 1% | | | |  |  |  |  |
| No-collaboration | 0% | | 0% | 0% | | | | 0% | | | |  |  |  |  |
| **Type of collaborations** | | | | | | | | | | | | | | |  |
| **Entity / Period** | **Government** | | | | **Private Org.** | | | | **Public Org.** | | | | **University** | |  |
|  | **2000-2010** | **2011-2021** | | | **2000-2010** | | **2011-2021** | | **2000-2010** | **2011-2021** | | | **2000-2010** | **2011-2021** |  |
| Government | 2% | 3% | | | 0% | 0% | | | 1% | 1% | | | 3% | 3% |  |
| Private Org. | 0% | 0% | | | 2% | 1% | | | 1% | 1% | | | 2% | 1% |  |
| Public Org. | 1% | 1% | | | 1% | 1% | | | 22% | 20% | | | 16% | 16% |  |
| University | 3% | 3% | | | 2% | 1% | | | 16% | 16% | | | 51% | 53% |  |

**Table A.4(b) Sectorial collaborations for patents**

| **Entity / Period** | **Individual** | | **Private Org.** | | **Public Org.** | | **University** | | **Government** | | **No-collaboration** | |
| --- | --- | --- | --- | --- | --- | --- | --- | --- | --- | --- | --- | --- |
|  | 2000-2010 | 2011-2021 | 2000-2010 | 2011-2021 | 2000-2010 | 2011-2021 | 2000-2010 | 2011-2021 | 2000-2010 | 2011-2021 | 2000-2010 | 2011-2021 |
| Individual | 4% | 1% | 18% | 2% | 3% | 0% | 0% | 0% | 0% | 0% | 7% | 36% |
| Private Org. | 0% | 0% | 5% | 2% | 0% | 1% | 0% | 5% | 1% | 0% | 42% | 27% |
| Public Org | 0% | 0% | 0% | 0% | 0% | 1% | 0% | 3% | 0% | 0% | 8% | 20% |
| University | 0% | 0% | 0% | 0% | 0% | 0% | 0% | 0% | 0% | 0% | 5% | 2% |
| Government | 0% | 0% | 1% | 0% | 0% | 0% | 0% | 0% | 0% | 0% | 6% | 1% |
| Government - Individual - Private Organization collaborations: 1% during 2000-2010 and 0% during 2011-2020  Government - Individual – University collaborations: 1% during 2000-2010 and 0% during 2011-2020 | | | | | | | | | | | | |

**Table A.5 (a) National and International Collaborative Efforts in Scientific Publishing**

|  | 2000-2010 | | | | | | 2011-2021 | | | |  |
| --- | --- | --- | --- | --- | --- | --- | --- | --- | --- | --- | --- |
| Country | | | **% of all Collabs** | | **% National Collabs** | **% Intern. Collabs** | **# of Collabs Countries** | **% of all Collabs** | **% National Collabs** | **% Intern. Collabs** | **# of Collabs Countries** |
| U.S. | | | | 21% | 85% | 15% | 33 | 19% | 82% | 18% | 74 |
| UK | | | | 6% | 69% | 31% | 32 | 5% | 61% | 39% | 60 |
| Spain | | | | 4% | 69% | 31% | 23 | 3% | 75% | 25% | 55 |
| Republic of Korea | | | | 5% | 91% | 9% | 8 | 2% | 90% | 10% | 31 |
| Japan | | | | 8% | 91% | 9% | 14 | 4% | 86% | 14% | 46 |
| Italy | | | | 3% | 83% | 17% | 24 | 3% | 79% | 21% | 54 |
| India | | | | 2% | 82% | 18% | 9 | 5% | 87% | 13% | 59 |
| Germany | | | | 10% | 80% | 20% | 28 | 7% | 74% | 26% | 66 |
| France | | | | 9% | 82% | 18% | 25 | 7% | 80% | 20% | 68 |
| China | | | | 7% | 89% | 11% | 18 | 19% | 90% | 10% | 59 |
| Canada | | | | 3% | 82% | 18% | 14 | 2% | 71% | 29% | 39 |
| Brazil | | | | 3% | 84% | 16% | 9 | 2% | 83% | 17% | 39 |
| Australia | | | | 2% | 78% | 22% | 21 | 3% | 74% | 26% | 53 |

**Table A.5 (b). Characteristics of Teams of Applicants and Inventors for Patents**

| **Country** | **Patents** | **Patents** | **Avg. Countries of Applicant /patent** | | **Avg. Applicants/Patent** | | **Avg. Inventors/Patent** | | **Avg. Countries of Inventor/patent** | |
| --- | --- | --- | --- | --- | --- | --- | --- | --- | --- | --- |
|  | **2000-2011** | **2010-2021** | **2000-2011** | **2010-2021** | **2000-2011** | **2010-2021** | **2000-2011** | **2010-2021** | **2000-2011** | **2010-2021** |
| United States | 54 | 94 | 2.07 | 1.53 | 1.00 | 1.02 | 3.83 | 4.12 | 1.09 | 1.07 |
| Belgium | 7 | 0 | 2.14 | - | 1.00 | - | 2.57 | - | 1.00 | - |
| Germany | 7 | 11 | 4.00 | 1.00 | 1.43 | 1.00 | 4.86 | 3.64 | 1.29 | 1.09 |
| India | 7 | 3 | 1.86 | 2.33 | 0.86 | 1.00 | 2.57 | 3.33 | 1.00 | 1.00 |
| Japan | 5 | 3 | 2.80 | 1.67 | 1.00 | 1.00 | 3.60 | 7.33 | 1.00 | 1.00 |
| China | 3 | 381 | 1.00 | 1.14 | 1.00 | 1.00 | 2.33 | 5.59 | 1.00 | 1.00 |
| Netherlands | 4 | 4 | 1.50 | 1.25 | 1.50 | 1.00 | 2.75 | 4.00 | 1.50 | 1.00 |
| Israel | 3 | 4 | 1.67 | 3.50 | 0.67 | 1.00 | 3.00 | 4.50 | 1.00 | 1.00 |
| Canada | 2 | 6 | 1.00 | 2.67 | 1.00 | 1.17 | 1.00 | 4.50 | 1.00 | 1.00 |
| Denmark | 2 | 0 | 1.00 | - | 1.00 | - | 4.50 | - | 1.00 | - |
| UK | 2 | 8 | 3.50 | 1.38 | 1.50 | 1.00 | 5.50 | 4.00 | 2.00 | 1.38 |
| Norway | 2 | 0 | 1.00 | - | 1.00 | - | 4.00 | - | 2.00 | - |
| South Korea | 1 | 7 | 1.00 | 1.00 | 1.00 | 1.00 | 3.00 | 6.43 | 1.00 | 1.00 |
| France | 1 | 6 | 1.00 | 2.17 | 1.00 | 1.00 | 3.00 | 5.17 | 1.00 | 1.00 |
| Switzerland | 1 | 6 | 1.00 | 1.00 | 1.00 | 1.00 | 4.00 | 2.17 | 1.00 | 1.00 |
| Taiwan | 0 | 4 | - | 1.00 | - | 1.00 | - | 1.50 | - | 1.00 |
| Australia | 1 | 2 | 2.00 | 3.50 | 1.00 | 1.50 | 4.00 | 3.00 | 1.00 | 1.50 |
| Portugal | 0 | 2 | - | 1 | - | 1 | - | 3.00 | - | 1.00 |
| Spain | 1 | 1 | 1.00 | 1.00 | 1.00 | 1.00 | 1.00 | 1.00 | 1.00 | 1.00 |
| Finland | 1 | 0 | - | - | - | - | 4.00 | - | 2.00 | - |
| Mexico | 0 | 1 | - | 1.00 | - | 1.00 | - | 9.00 | - | 1.00 |
| Singapore | 0 | 1 | - | 1.00 | - | 1.00 | - | 1.00 | - | 1.00 |
| Czech Republic | 0 | 1 | - | 1.00 | - | 1.00 | - | 3.00 | - | 1.00 |
| Colombia | 0 | 1 | - | 2.00 | - | 1.00 | - | 2.00 | - | 2.00 |
| Argentina | 0 | 1 | - | 5.00 | - | 2.00 | - | 3.00 | - | 1.00 |

**Appendix B**

**Query syntax EspaceNet for Patent Data Collection**

We queried EspaceNet using the following syntax, where ctxt defines the fields in which the key terms were searched, which included patent title, abstract and claim: *(ctxt all "plant phenotyp*" OR ctxt all "crop phenotyp*" OR ctxt all "plant phenomic*" OR ctxt all "crop phenomic*") AND (ctxt all "high throughput" OR ctxt all "digital" OR ctxt all "imag*" OR ctxt all "automat*" OR ctxt all "robotic*" OR ctxt all "infrared").* EspaceNet only allows 10 terms to be queried at once, so we ran some subsequent searches that included other terms – “aeronautics”, “Minimum Information About a Plant Phenotyping Experiment”, “MIAPPE”, “thermography”, “big data”, however no additional patents were identified.

**Search Terms and Criteria for Publication Data Collection**

WoS**:** ((((TI=((Plant* OR crop*) AND (Phenotyp* OR phenomic*) )) OR (AK=((Plant* OR crop*) AND (Phenotyp* OR phenomic*) )) OR (KP=((Plant* OR crop*) AND (Phenotyp* OR phenomic*) ) OR (AB=((Plant* OR crop*) AND(Phenotyp* OR phenomic*) )) NOT (TS =human*) )))))  AND LANGUAGE:(English) AND DOCUMENT TYPES:(Article OR Book Chapter OR Data Paper OR Early Access OR Editorial Material OR Letter OR Proceedings Paper OR Review) Indexes=SCI-EXPANDED, SSCI, A&HCI, CPCI-S, CPCI-SSH, BKCI-S, BKCI-SSH, ESCI, CCR-EXPANDED, IC Timespan=2000-2021

SCOPUS**:** ( ABS ( ( plant*  OR  crop* )  AND  ( phenotyp*  OR  phenomic* ) )  OR  KEY ( ( plant*  OR  crop* )  AND  ( phenotyp*  OR  phenomic* ) )  OR  TITLE ( ( plant*  OR  crop* )  AND  ( phenotyp*  OR  phenomic* ) ) )  AND  ( LIMIT-TO ( PUBYEAR ,  2021 )  OR  LIMIT-TO ( PUBYEAR ,  2020 )  OR  LIMIT-TO ( PUBYEAR ,  2019 )  OR  LIMIT-TO ( PUBYEAR ,  2018 )  OR  LIMIT-TO ( PUBYEAR ,  2017 )  OR  LIMIT-TO ( PUBYEAR ,  2016 )  OR  LIMIT-TO ( PUBYEAR ,  2015 )  OR  LIMIT-TO ( PUBYEAR ,  2014 )  OR  LIMIT-TO ( PUBYEAR ,  2013 )  OR  LIMIT-TO ( PUBYEAR ,  2012 )  OR  LIMIT-TO ( PUBYEAR ,  2011 )  OR  LIMIT-TO ( PUBYEAR ,  2010 )  OR  LIMIT-TO ( PUBYEAR ,  2009 )  OR  LIMIT-TO ( PUBYEAR ,  2008 )  OR  LIMIT-TO ( PUBYEAR ,  2007 )  OR  LIMIT-TO ( PUBYEAR ,  2006 )  OR  LIMIT-TO ( PUBYEAR ,  2005 )  OR  LIMIT-TO ( PUBYEAR ,  2004 )  OR  LIMIT-TO ( PUBYEAR ,  2003 )  OR  LIMIT-TO ( PUBYEAR ,  2002 )  OR  LIMIT-TO ( PUBYEAR ,  2001 )  OR  LIMIT-TO ( PUBYEAR ,  2000 ) )  AND  ( LIMIT-TO ( LANGUAGE ,  "English" ) )  AND  ( LIMIT-TO ( EXACTKEYWORD ,  "Nonhuman" ) )  AND  ( LIMIT-TO ( DOCTYPE ,  "ar" )  OR  LIMIT-TO ( DOCTYPE ,  "re" )  OR  LIMIT-TO ( DOCTYPE ,  "ch" )  OR  LIMIT-TO ( DOCTYPE ,  "cp" )  OR  LIMIT-TO ( DOCTYPE ,  "sh" )  OR  LIMIT-TO ( DOCTYPE ,  "ed" )  OR  LIMIT-TO ( DOCTYPE ,  "le" )  OR  LIMIT-TO ( DOCTYPE ,  "dp" ) )

**Screening Steps for scientific Publication Data**

Our search within the two academic databases consisted of equivalent search terms, operands, and fields. In WoS we looked for records with the above keywords specified in the title of the record, or the abstract, or author keywords or keyword plus. In WoS, these fields of a publication are captured under the topic search criteria, hence the TS in the search query string above. In SCOPUS we searched within “Abstract, Title and Keywords”.  Two datasets were obtained, one containing a total of 41,337 records retrieved from WoS and the second one with 16,866 records, retrieved from SCOPUS. Each record contained several metadata fields (e.g., record title, abstract, keywords, author affiliation, etc.).

The next steps in processing the collected data involved merging the two databases and the elimination of duplicated records. This was done using the function *mergeBdSource* from the Bibliometrix R Package. *The mergeBdSource function formed based on the intrinsic R function “duplicated”.* The function was used to merge the two datasets, and to eliminate duplicates. The merge of the two datasets from SCOPUS and WoS, resulted in a total number of 48,978 unique records. These records were in the broader area of agricultural phenotyping. In order to only screen into the final dataset records on digital agricultural phenotyping, we applied a query on this large dataset, and searched the records titles, abstract and key words, retaining only the ones containing one of the words: *"high throughput", "digital", "imag*", "automat*", "robotic*", "infrared",* “aeronautics”, “Minimum Information About a Plant Phenotyping Experiment”, “MIAPPE”, “thermography”, “big data”. This returned a total number of 7,732 records.

The final step involved screening the records, by reading the title and skimming the abstract if deemed necessary, in order to ensure that all records included articles of interest. In this process, 559 records were discarded, leaving 7,173 records in the final dataset.
